# Estimation of Excess Mortality in Germany During 2020-2022

**DOI:** 10.7759/cureus.39371

**Published:** 2023-05-23

**Authors:** Christof Kuhbandner, Matthias Reitzner

**Affiliations:** 1 Department of Human Sciences, University of Regensburg, Regensburg, DEU; 2 Institute for Mathematics, University of Osnabrück, Osnabrück, DEU

**Keywords:** causes of deaths, mortality trends, expected mortality, covid-19, excess mortality

## Abstract

Background

This study estimates the burden of COVID-19 on mortality in Germany. It is expected that many people have died because of the new COVID-19 virus who otherwise would not have died. Estimating the burden of the COVID-19 pandemic on mortality by the number of officially reported COVID-19-related deaths has been proven to be difficult due to several reasons. Because of this, a better approach, which has been used in many studies, is to estimate the burden of the COVID-19 pandemic by calculating the excess mortality for the pandemic years. An advantage of such an approach is that additional negative impacts of a pandemic on mortality are covered as well, such as a possible pandemic-induced strain on the healthcare system.

Methods

To calculate the excess mortality in Germany for the pandemic years 2020 to 2022, we compare the reported number of all-cause deaths (i.e., the number of deaths independently of underlying causes) with the number of statistically expected all-cause deaths. For this, the state-of-the-art method of actuarial science, based on population tables, life tables, and longevity trends, is used to estimate the expected number of all-cause deaths from 2020 to 2022 if there had been no pandemic.

Results

The results show that the observed number of deaths in 2020 was close to the expected number with respect to the empirical standard deviation; approximately 4,000 excess deaths occurred. By contrast, in 2021, the observed number of deaths was two empirical standard deviations above the expected number and even more than four times the empirical standard deviation in 2022. In total, the number of excess deaths in the year 2021 is about 34,000 and in 2022 about 66,000 deaths, yielding a cumulated 100,000 excess deaths in both years. The high excess mortality in 2021 and 2022 was mainly due to an increase in deaths in the age groups between 15 and 79 years and started to accumulate only from April 2021 onward. A similar mortality pattern was observed for stillbirths with an increase of about 9.4% in the second quarter and 19.4% in the fourth quarter of the year 2021 compared to previous years.

Conclusions

These findings indicate that something must have happened in spring 2021 that led to a sudden and sustained increase in mortality, although no such effects on mortality had been observed during the early COVID-19 pandemic so far. Possible influencing factors are explored in the discussion.

## Introduction

In the last two years, the burden of the COVID-19 pandemic on mortality has been intensively discussed. Since COVID-19 is an infectious disease that is caused by a new virus, it is expected that many people have died because of the new virus who otherwise would not have died. This expectation represents one of the central justifications for the taking of countermeasures against the spread of the virus. Due to this reason, several previous studies have tried to estimate the extent of the mortality burden that has been brought about by the COVID-19 pandemic.

At first glance, it seems obvious to simply estimate the burden of the COVID-19 pandemic on mortality based on the number of officially reported COVID-19-related deaths. However, this has been proven to be difficult due to several reasons.

First, it is not specified whether a reported COVID-19 death was indeed caused by a SARS-CoV-2 infection or whether the deceased person died from some other cause of death with a coincidentally occurring SARS-CoV-2 infection.

There is evidence that the diagnostic problem of counting deaths as COVID-19 deaths although they were not caused by COVID-19 was particularly severe in later phases of the pandemic. For instance, a study from Denmark [1] showed that in 2022, about 70% of the reported COVID-19 deaths were not caused by a SARS-CoV-2 infection. A published analysis [2] of the German COVID-19 autopsy registry showed that until October 2021, at least 14% of the reported COVID-19 deaths were not caused by a SARS-CoV-2 infection. Hence, estimating the burden of the pandemic based on reported COVID-19 deaths may overestimate the true burden due to the erroneous counting of deaths as COVID deaths, which were caused due to other reasons.

Second, even if a person died from COVID-19, this does not rule out the possibility that the person would have died as well even if there had been no COVID-19 pandemic, for example, because of a rhinovirus infection [3] or just because they were highly frail [4]. Accordingly, even if there is a large number of deaths that were caused by a SARS-CoV-2 infection, this would not necessarily mean that all these deaths are additional that would not have occurred if there had been no COVID-19 pandemic.

All-cause mortality: estimating the burden of the COVID-19 pandemic

An obvious way to solve such problems when estimating the burden of the COVID-19 pandemic on mortality is to compare the number of observed all-cause deaths independently of the underlying causes of deaths with the number of all-cause deaths that would have been expected if there had been no pandemic. If there is a new virus that causes additional deaths beyond what is usually expected, the number of observed all-cause deaths should be larger than the number of usually expected deaths, and the higher the number of observed deaths is above the number of usually expected deaths, the higher is the burden of a pandemic on mortality. In particular, beyond the advantage that the aforementioned problems with the number of reported COVID-19-related deaths are avoided, another advantage is that additional indirect negative impacts of a pandemic on mortality are covered as well, such as a possible pandemic-induced strain on the healthcare system.

Therefore, it is not surprising that several attempts have been made to estimate the increase in all-cause mortality during the COVID-19 pandemic [5-11]. As the death of a person is a clear diagnostic fact and highly reliable data on mortality are available for several countries, one may expect that the question of whether more people have died than is usually expected can be answered.

However, the existing attempts show very large differences in the estimated increase in all-cause mortality during the COVID-19 pandemic. This can be illustrated in Germany where highly reliable data on the number of all-cause deaths even at the level of individual days are available. The estimated increase in all-cause mortality during the pandemic years 2020 and 2021 varies from 203,000 additional deaths [6] to only 29,716 additional deaths [7,8], and for the pandemic year 2020, it has even been estimated that less number of all-cause deaths have been observed than usually expected [9].

How can this large variability in the estimated increase in all-cause mortality be explained? The number of observed all-cause deaths is clearly defined (although it seems that even in Germany it is difficult to determine this number precisely). But the estimation of the usually expected deaths is relatively complex and entails several choices of mathematical models and parameters and which can lead to large differences in the estimated values.

Against this background, this study has the objective to provide a best practice method, the state-of-the-art method of actuarial science, to estimate the expected all-cause mortality using the example of all-cause deaths in Germany in the years 2020 to 2022. The underlying standard model in actuarial mathematics was already used by Euler and Gauß, modern developments take into account mortality trends and longevity factors. Using this method, the increase in all-cause mortality in Germany for the pandemic years 2020 to 2022 is estimated. In addition, an evaluation of the model and parameter choices that must be made is provided. This demonstrates that the amount of increase in all-cause mortality varies depending on the chosen model and parameters.

As described earlier, several studies have attempted to estimate the increase in mortality in Germany in 2020, 2021, and 2022 based on different methods [6-9,11]. However, there are several unanswered questions.

Only one study [6] in 2020 took into account the historical trend in mortality rates. We use the mathematical model provided by the German Association of Actuaries. This includes longevity factors, which are well-established in actuarial science.

Although in most of the studies, age-standardized estimations were made, age-dependent differences in mortality increase or decrease were not examined in detail. We use the most recent life tables of the Federal Statistical Office of Germany to calculate age-dependent expectations.

In none of the previous studies, it was examined how much the mortality estimates depend on the underlying data and vary with different approaches. We state the data uncertainty and calculate the model and parameter sensitivity by comparing the results achieved using different life tables and longevity factors.

In all of the previous studies except a recent study [12] concerning Austria, only the estimated increase in all-cause deaths was reported, without examining whether the estimated increase exceeds the usual variation in mortality found across previous years. Even in the most recent study of Levitt et al. [13] where a multiverse analysis approach was used that considered several different periods for defining the reference baseline used to estimate the number of expected deaths, only the variation in excess mortality estimates across different reference baselines was reported, but the variation in mortality across the years used as the reference baseline was not examined. We estimate the yearly empirical standard deviation, which could be used to obtain confidence intervals.

The increase in mortality over the year has so far only been investigated for 2020 in two studies [6,7] and for 2021 in one study [8]. The year 2022 has not yet been investigated in this respect. Furthermore, no study has yet determined the increase in mortality over the year for different age groups.

In none of the previous studies, possible factors that might contribute to the observed course of the increase in mortality were explicitly examined on a monthly base during the pandemic years 2020 to 2022.

In all previous studies, the increase in mortality has only been examined for the age groups 0 and above. Whether changes in mortality are also found at the level of stillbirths has not been investigated so far.

As will be shown, a proper analysis of the increase in all-cause mortality reveals several previously unknown dynamics that will require a reassessment of the mortality burden brought about by the COVID-19 pandemic.

This article was previously posted to the ResearchGate preprint server on February 3, 2023.

Estimating the increase in all-cause mortality: population size and historical trend effects

Two main effects have to be taken into account when estimating the increase in all-cause mortality: the effects of changes in the size and age profile of the population and the effects of historical trends in mortality rates.

Changes in population size and age profile have to be taken into account due to the simple fact that the larger or older a population is, the more deaths occur. Regarding the population over 80 years of age in Germany, its size, and thus the number of deaths, increases from year to year. Concluding from this pattern that mortality increased in the years 2020 and 2021 compared to previous years would make no sense because this increase is fully attributable to the increase in population size.

Historical trends in mortality rates have to be taken into account because mortality rates are influenced by environmental and societal changes and improvements in medical treatments. In Germany, there is a historical trend of a continuous decrease in the mortality rate that is observed in most age groups. If such a declining trend in mortality rates is not taken into account, the number of expected deaths is overestimated, and thus, the true mortality excess is underestimated.

The pitfall of ignoring changes in population size is for example found in the estimations provided by the German Federal Statistical Office [14] where the increase in mortality is estimated based on a comparison of the observed number of deaths with the median value of the four previous years. Estimating the number of expected deaths based on the median of the four previous years underestimates the number of expected deaths and thus overestimates the true increase in mortality. The invalidity of this method can be illustrated by the fact that in case of a continuously increasing population size, as is the case for the population over 80 years of age in Germany, such a method would conclude for *every* year that there was an unexpected increase in mortality compared to previous years.

The pitfall of ignoring longer historic trends is for example found in the estimations provided by the World Health Organization (WHO) [11] where the increase in mortality is estimated based on a thin-plate spline extrapolation of the number of observed deaths. Such an estimation method is highly sensitive to short-term changes in the observed number of deaths, erratic estimations of expected deaths predictions can occur. Regarding the WHO estimations for Germany, the spline extrapolation predicts - based on the short-term decline in deaths in 2019 compared to 2018 - that a similar decline would occur in the following years as well, although this completely contradicts the long-term historical trend. The estimations provided by the WHO not only ignore long-term trends but also changes in population. This will be discussed in the next section.

Methods that take into account population size and historical trends effects

A first and comparatively simple approach to take into account population size and historical trends effects is the attempt to predict the further course of the number of deaths from observed data in previous years using regression methods. In a study by Baum [5], the course of the observed increase in the number of deaths in Germany from 2001 to 2021 compared to the year 2000 was fitted with a polynomial function of order two, and the yearly residuals were used to estimate the yearly increase or decrease in mortality, resulting in an estimated increase in mortality in the years 2020 and 2021 of about 11,000 additional deaths each. While the advantage of this approach is, on the one hand, that no parameter choices have to be made, on the other hand, this is, at the same time, the weakness of this approach: as every data point is given the same weight, unique outliers may lead to biased estimations, and developments depending on more complex circumstances cannot be incorporated in this approach.

To account for unique outliers, it has been tried to estimate the number of expected deaths by a time-series model based on the number of observed deaths in previous years and to exclude past phases of unique excess mortality, as done in the European Mortality Monitoring (EuroMOMO) project [15]. Beyond the problem that the resulting estimates depend on the specific model and parameter choices made, a common problem for every approach that bases estimations on the raw number of observed deaths is that the resulting estimations do not take into account possible changes in the age structure within a population, which can lead to biased estimates.

To take into account the age structure within a population, so-called age adjustments have a long tradition in mortality research [16], which is essential, especially when estimating the number of expected deaths in populations where the proportion of elderly people changes over time. The basic method is to compute mortality rates for a reference period separately for different age groups and to extrapolate from the age-dependent mortality rates and the population sizes of the different age groups in the to-be-estimated year the number of expected deaths in each of the age groups.

In a recent study by Levitt et al. [10], the increase in mortality in the years 2020 and 2021 was estimated based on the reference period of the three pre-pandemic years 2017-2019 using age strata of 0-14, 15-64, 65-74, 75-84, and 85+ years, resulting in an estimated increase in mortality of about 16,000 additional deaths in the year 2020 and 38,800 additional deaths in the year 2021. In two studies by De Nicola et al. [7,8], a more refined (see below) and more fine-grained age adjustment method was used, resulting in even lower estimates of increased mortality, with about 6,300 additional deaths in 2020 and 23,400 additional deaths in 2021.

A problem in both the study by Levitt et al. [10] and the studies by De Nicola et al. [7,8] is that possible historical trends in mortality rates are not taken into account. This was, in addition to an age adjustment, done in a study by Kowall et al. [9] where the increase in mortality in the year 2020 was estimated for the countries Germany, Spain, and Sweden. Historical trends in mortality rates were estimated based on the observed decrease in mortality rates in the pre-pandemic years 2016-2019. For Germany, it was estimated that the number of observed deaths in 2020 was 0.9% higher than the number of estimated expected deaths, which is in the range of the estimations in the study by De Nicola et al. [7,8]. Estimations with adjustments for changes in historical trends in mortality rates for the years 2021 and 2022 have not been reported to date, at least to our knowledge.

The inherent model uncertainty of estimates of increases in mortality

As has already become apparent in the previous sections, the estimation of the amount of increase in all-cause mortality entails several model and parameter choices that have to be made. While a proper analysis necessarily requires taking into account changes in population sizes and historical trends in mortality rates, there remain several degrees of freedom in how to exactly do this. For instance, an open question is which previous years are used as a reference and which model is used for the extrapolation of the expected deaths based on these years.

First, when reporting estimates of the amount of increase in mortality, it is important to show how strongly the estimates vary with different model and parameter choices. Possible choices and the resulting estimates should be communicated to readers in a way so that they are enabled to draw their conclusions depending on the specific questions they would like to answer.

Second, when interpreting estimates of the increase in mortality, one has to be aware of the model and parameter choices. When deciding which approach is chosen, one has to clarify which question one tries to answer, and choose the approach that best fits the to-be-answered question. For instance, if one is interested in the question of how far the observed number of deaths is above the usually occurring deaths, excluding outlier years when estimating the amount of increase in mortality may be a reasonable decision. However, if one is interested in whether the observed number of deaths is above the extreme values of previous years, excluding outliers may be a less reasonable decision.

Third, despite the inherent uncertainty of the estimates of increases in mortality, differences in mortality increase between different periods or different regions are largely robust against the parameter and model choice. This was recently shown in a study by Levitt et al. [13] where a so-called multiverse analysis approach was used that considered different periods for defining the reference baseline on which the estimation of the expected number of deaths was based. While there was large variability in the absolute magnitude of the different excess mortality estimates, the relative ranking of different countries compared to others remained largely unaltered across the different reference baselines. Furthermore, regarding the assessment of the magnitude of estimated excess mortality, if the observed difference in estimations between several parameter choices is small compared to the empirical standard deviation occurring in previous years, an observed number of deaths that is several standard deviations above the estimated expected number of deaths can be assumed to reflect the fact that mortality increased considerably.

The use of the term *excess mortality*

In many of the previous studies, the observation that the number of observed all-cause deaths is larger than the number of expected all-cause deaths is designated by the term *excess mortality*. Such use of terms is questionable. The number of deaths from year to year does not follow a straight line but varies around a common trend. If one were to designate *excess mortality year *as all years in which more deaths are observed than expected according to the common trend, one would have to conclude that *excess mortality* is observed in about 50% of all years and a *mortality deficit* in the other 50% of all years.

As about half of the years show mortality levels above the common trend, one could use the term *excess mortality* only for years that show an outstanding increase in mortality above a certain threshold. One straightforward possibility to establish such a threshold would be to compute the mean variation (empirical standard deviation) around the common trend across the years and to designate as *years of significant excess mortality*, only those in which the number of observed deaths exceeds twice the mean variation.

Another possibility would be to search for previous years with peak deviations from the common trend and then compare the deviation observed in the year one is interested in with the peak deviations in previous years. Such a comparison was, for instance, made in a recent study by Staub et al. [17] where the historical dimension of the COVID-19 pandemic was examined for the countries Switzerland, Sweden, and Spain over more than 100 years, revealing that the peaks of monthly excess mortality in 2020 were greater than most peaks since 1918.

Nevertheless, also in this contribution, we decided to use the terms *excess mortality* and *mortality deficit* for mortality, which is just above and below, respectively, the estimated value, as in most other contributions. An attempt to define an outstanding *excess mortality year* via mean variations will be made in the sections on data uncertainty and the empirical standard deviation.

## Materials and methods

Yearly expected mortality

The standard method in actuarial science uses life tables and population tables to obtain the expected number of deaths. Historical population tables are used to estimate the longevity trend, which is taken into account.

Thus, the starting point for our investigations is the period life tables and population demographics available from the Federal Statistical Office of Germany. As usual in actuarial science, we denote by $l_{x,t}$ the number of $x$-year-old males on January 1 in year $t$, by $l_{y,t}$ the number of $y$-year-old females on January 1 in year $t$, by $d_{x,t}$ the number of deaths of $x$-year-old males in year $t$, by $d_{y,t}$ the number of deaths of $y$-year-old females in year $t$, by $q_{x,t}$ (an estimate for) the mortality probability for an $x$-year-old male in year $t$, and by $q_{y,t}$ (an estimate for) the mortality probability for a $y$-year-old female in year $t$. Note that $d_{x,t}$ also contains deceased who have been $(x-1)$ years old on January 1 in year $t$ and died as $x$ year old. The method of Farr is a standard tool to take this age shift into account. Also, the 2017/2019 life table of the Federal Statistical Office of Germany [18] uses Farr's approach to estimate $q_{x, t}$ and $q_{y, t}$ by the point estimates $\hat q_{x,t}$ and $\hat q_{y,t}$,

\begin{equation}\hat q_{x,2019} = \frac {\sum_{t=2017}^{2019}\ d_{x,t}}{\frac12 \sum_{t=2017}^{2019}\ (l_{x,t} + l_{x,t+1})+ \frac 12 \sum_{t=2017}^{2019}\ d_{x,t}}\end{equation}

and analogously $\hat q_{y,2019}$.

The period life table 2017/2019 of the Federal Statistical Office of Germany [18] thus takes into account the mortality in three years. It contains the mortality probabilities $\hat q_{x,2019}\,$ and $\hat q_{y,2019}\,$, and the underlying population table the population size $l_{x,t}$ and $l_{y,t}$ for the age $x,y=0, \dots, 100$. In principle, it would be more precise to use life tables and population tables up to age 113, but these data are not available.

A much more complicated task is to estimate the historical trend, which yields generation life tables. Generation life tables observe the mortality development over a long period, roughly 100 years, smoothen the existing data, and estimate the historical trend of the mortality probabilities. These probabilities have been decreasing within the last 100 years. The common ansatz is to fix some base year $t_0$ and to set \begin{equation} q_{x,t}= q_{x, t_0}\, e^{- F(x; t,t_0)} , \ q_{y,t}= q_{y, t_0}\, e^{- F(y; t,t_0)} . \end{equation} As it is well known that mortality probabilities for males and females differ substantially, these two cases are computed separately. The German Association of Actuaries (Deutsche Aktuarvereinigung, or DAV, in German) recommends using a smoothed life table $q_{x, t_0}$ in a base year $t_0$ and to model the trend underlying future mortality, the longevity trend function $F(x; t,t_0)$, via regression separately for the male and female population. In the year 2004, it turned out that the decrease in the mortality probabilities in the previous years has been steeper than expected; therefore, the DAV life table DAV 2004 R [19] distinguishes between a higher short-term trend and a lower long-term trend. These trends are used for life annuities, whereas for life insurance the trend (at least the short-term trend) is mostly ignored.

One should keep in mind that the life tables DAV 2004 R and the longevity factors DAV 2004 R are tailor-made for pension funds. As we are interested in predictions concerning the whole German population, we use the life table for the general population of the Federal Statistical Office of Germany, not the life table DAV 2004 R, and adapt the longevity factors of the DAV 2004 R to fit the whole population. In addition, it seems that the longevity trend was flattening in the last few years. Therefore, we have decided to use half the long-term trend function given by DAV 2004 R, \begin{equation} F(x; t,t_0)= \frac 12 (t-2019) F_{l,x} ,\ F(y; t,t_0)= \frac 12 (t-2019) F_{l,y}\end{equation} where the numbers $F_{l,x}$ and $F_{l,y}$ are contained in the DAV 2004 R table. We will use the point estimates $\hat q_{x,2019}$ and $\hat q_{y,2019}$ from the last pre-pandemic life table 2017/2019 by the Federal Statistical Office of Germany as the base life table in a first step and thus take $t_0=2019$. Another possible choice would be to take $t_0=2018$, the mean year of the table, which only results in minor changes, but we follow the actuarial standard to set $t_0$ as the year when the table was completed. We also will compare the obtained results to the ones obtained using the previous life tables 2015/2016 and 2016/2017, then clearly with $t_0=2016$ and $t_0=2017$, respectively.

We remark that modeling the longevity factors is a challenging task. For example, the need for longevity factors depends heavily on the country; it seems that in Japan and England, the decrease of the mortality trend has already stopped, that is, $F(x;t,t_0)=F(y;t,t_0)=0$, and mortality probabilities are (more or less) constant. For a discussion concerning our model parameters, that is, our choice of using half of the longevity trend and the (nonsmoothed) life table 2017/2019, we refer to the next section.

Putting things together, we define the mortality probability of an $x$-year-old male in year $t$ by \begin{equation}q_{x,t} = \hat q_{x, 2019} e^{- \frac 12 (t-2019) F_{l,x}},\end{equation} and for a $y$-year-old female in year $t$ by \begin{equation}q_{y,t} = \hat q_{y, 2019} e^{- \frac 12 (t-2019) F_{l,y}}.\end{equation}

Now, for each individual, the probability to die at age $x$ years is given by $q_{x,t}$, and hence, in a first attempt, a population of $l_{x,t}$ individuals produces binomial distributed random numbers $D_{x,t}$ and $D_{y,t}$ of deaths for males and females, respectively, with expected values \begin{equation}\mathbb E D_{x,t}= l_{x,t} q_{x,t}, \mathbb E D_{y,t}= l_{y,t} q_{y,t}. \end{equation} As is already discussed earlier in connection with Farr's method, this formula ignores those individuals who have been of age $(x-1)$ years at the beginning of year $t$ and died as $x$-year-old. To compensate for this missing piece, we follow the procedure proposed by De Nicola et al. [7]. Roughly half of the $x-1$-year-old population at the beginning of the year, which is of size $l_{x-1,t}$, dies after their birthday as $x$-year-old. For them, we use the smoothed mortality probability \begin{equation}\frac{q_{x-1,t}+q_{x,t}}{2} .\end{equation} The other half of the $x$-year-old deceased belongs to the population of $x$-year-old at the beginning of the year, which is of size $l_{x,t}$. For them, we use the smoothed mortality probability \begin{equation}\frac{q_{x,t}+q_{x+1,t}}{2} .\end{equation} For more details, see [7]. Hence, for $x=0, \dots,\,101$, the random number $D_{x,t}$ of deaths of age $x$ in year $t=2020,\, 2021,\, 2022$, is binomially distributed and satisfies \begin{equation}\mathbb E D_{x,t} = \frac12 \left( l_{x-1,t} \frac{q_{x-1,t}+q_{x,t}}{2} + l_{x,t} \frac{q_{x,t}+q_{x+1,t}}{2} \right) \end{equation} and an analogous formula holds for $\mathbb E D_{y,t}$. Here, $l_{x-1,t}$ and $l_{x,t}$ are taken from the population table of the Federal Statistical Office of Germany [20]. Note that the reported number of deaths is from the most current data set of the Federal Statistical Office of Germany; the data set for 2022 is still preliminary, and there will be changes within the next months. For $x=0$, we set $l_{-1, t} = l_{0, t+1}$ if available, else $l_{-1, t} = l_{0, t}$ and $q_{-1, t} = q_{0, t}$. The same considerations lead to $\mathbb E D_{y,t}$.

Let us denote by $d_{x,t}$ and $d_{y,t}$, respectively, the observed number of deaths of $x$-year-old males and $y$-year-old females in year $t$, and put \begin{equation} d_{a,t}= \sum_{x \in a} d_{x,t} + \sum_{y \in a} d_{y,t}\end{equation} for some age group $a$. The Federal Statistical Office of Germany [21] offers tables for the observed number of male deaths and female deaths for the age groups \begin{equation} a\in\{0\text{-}14, 15\text{-}29, 30\text{-}34, 35\text{-}39,\dots, 90\text{-}94, 95+ \} \end{equation} which we use for the years $t=2020,\, 2021,\, 2022$. The excess mortality is obtained by comparing the expected values \begin{equation} \mathbb E D_{a,t} = \sum_{x \in a} \mathbb E D_{x,t} + \sum_{y \in a} \mathbb E D_{y,t} \end{equation} to the observed data $d_{a,t}$ for each age group and $t=2020,\, 2021$, and $2022$. Because the year 2020 is a leap year, we have added a day by multiplying the result of the computations described above by $\frac{366}{365}$.

The mortality probabilities differ for the male and female populations. However, the excess mortality is nearly the same for the male and female population. Hence, we calculate the expected number of deaths separately and show only the total number of deaths. On the other hand, huge differences occur for the excess mortality in different age groups, and therefore, we will present our results for each age group separately. The mortality probability also depends significantly on social status, profession, health condition, region, etc. The German life tables give average mortality probabilities. It is unclear - at least to the authors - whether the SARS-CoV-2 infection rate and mortality depend on these factors, too. For a deeper investigation of the COVID-19 mortality increase, this should be taken into account, but appropriate data are not available.

Data uncertainty and fluctuations

Having modeled the number of deaths as a binomial random variable and computed the expectation and the excess mortality, it would be desirable to state a confidence interval to judge whether we observe the usual excess mortality in the pandemic years or whether excess mortality is beyond the expected fluctuations. Yet when presenting our results it will turn out that the historically observed fluctuations are much larger than the fluctuation of a binomial random variable. Because of this limitation of our mathematical model, we avoid the use of the words confidence interval for excess mortality. Nevertheless, in this section, we will generate estimates for the data uncertainty concerning $\hat q_{x,t}, \hat q_{y,t}$ and the longevity factors $F(x;t,t_0), F(y;t,t_0)$ and calculate a simple estimate for the empirically observed standard deviation of $D_{x,t}, D_{y,t}$. The obtained estimates for the model and data uncertainty and the empirical standard deviation then enable the reader to compare the observed excess deaths in the pandemic years 2020, 2021, and 2022 to the inherent data uncertainty and historical fluctuations.

First, the most basic data set for estimating excess mortality is the number of all-cause deaths each year. Each week, the Federal Statistical Office of Germany publishes the number of reported deaths. After the end of the year, the Federal Statistical Office of Germany undertakes a plausibility check and then publishes the corrected final number of deaths about September next year. For instance, for 2019, this resulted in a change of at least 20,000 data sets, yielding a cumulative change of nearly 3,000 deaths, and for 2021, we observe a cumulative change of more than 2,000 deaths. Hence, even in a country like Germany, already the number of observed deaths seems to have an intrinsic uncertainty of 2,000 to 3,000 deaths. Also, there is an inherent uncertainty in the population size and age distribution published by the Federal Statistical Office. These tables are based on the 2011 German census and updated from year to year using some prediction model.

Second, the life tables and the use of the longevity factors for modeling mortality probabilities essentially influence the results. One could replace the 2017/2019 life table of the Federal Statistical Office of Germany with the life tables 2016/2018 or 2015/2017. One could use different longevity factors or ignore them completely. The answer to the question, of whether serious excess mortality occurred for 2020, 2021, and 2022, heavily depends on these underlying data sets. For a sensitivity analysis concerning this model assumptions, we will present the total expected number of deaths \begin{equation} \mathbb E D_{t} = \sum_{x=0}^{101} \mathbb E D_{x,t} + \sum_{y=0}^{101} \mathbb E D_{y,t} \end{equation} and the excess mortality for different life tables and taking into account either none, half, or the full longevity trend. The difference yields an estimate for the model uncertainty due to the choice of the life table and the longevity factor.

Third, we are interested in a rough approximation of the empirical standard deviation. We use an extremely simple model, a linear regression for the observed number of deaths \begin{equation} d_{t} = \sum_{x=0}^{100} d_{x,t} + \sum_{x=0}^{100} d_{y,t} \approx L(t) = \alpha + \beta (t-2009) , \end{equation} for $t=2010, \dots, 2019$ and calculate the empirical standard deviation $\hat \sigma (d_{t})$ in this model. We take into account that 2012 and 2016 were leap years. The same method, a linear regression model, can be applied to age groups $a$, estimating the observed empirical variance $\hat \sigma (d_{a,t})$.

Monthly expected mortality

Having computed the yearly excess mortality, we investigate in more detail the number of deaths during the years 2020 to 2022. It is well known that the mortality probabilities differ from month to month with possible peaks in winter and also sometimes in summer when the weather is too hot.

Unfortunately, the data basis for such investigations provided by the Federal Statistical Office of Germany is somehow weak, so we have to apply several approximation steps. Let us denote the observed number of deaths of $x$-year-old males and $y$-year-old females, respectively, by by $d_{x,t,m}$ and $d_{y,t,m}$ in year $t$ and in month $m$. The Federal Statistical Office of Germany offers tables for the observed number of male deaths and female deaths for the age groups \begin{equation} a\in\{0\text{-}14, 15\text{-}29, 30\text{-}34,35\text{-}39,\dots,90\text{-}94,95+\} \end{equation} which we use for the years $t=2010, \dots, 2022$ [21].

For $x \in a$, denote the estimated proportion of male deaths in month $m$, $m=1, \dots, 12$ by \begin{equation} f_{x, m} = \frac 1{10} \sum_{t=2010}^{2019} \frac{\sum_{x \in a}d_{x,t,m}}{\sum_{x \in a} d_{x,t}}, \sum\limits_{m=1}^{12} f_{x, m} =1 , \end{equation} where we consider that 2012 and 2016 were leap years. The results for the male deaths are given in Table 1.

**Table 1:** Estimated proportion of male deaths in month $m$ .

Age\month	1	2	3	4	5	6	7	8	9	10	11	12
0-14 years	8.8%	8.1%	9.1%	8.3%	8.1%	8.5%	8.5%	8.5%	7.9%	8.1%	7.8%	8.4%
15-29 years	8.5%	7.6%	8.3%	8.3%	8.7%	8.6%	9.2%	8.7%	8.2%	8.3%	8.0%	7.7%
30-34 years	8.7%	7.7%	8.6%	8.5%	8.6%	8.4%	8.8%	8.5%	7.7%	8.4%	8.0%	8.1%
35-39 years	8.3%	7.9%	8.8%	8.2%	8.7%	8.1%	8.7%	8.6%	7.8%	8.3%	7.9%	8.8%
40-44 years	8.9%	8.3%	9.1%	8.4%	8.4%	8.0%	8.4%	8.3%	8.1%	8.1%	8.0%	8.1%
45-49 years	9.2%	8.2%	8.9%	8.3%	8.3%	8.2%	8.4%	8.2%	7.9%	8.2%	7.9%	8.2%
50-54 years	9.0%	8.2%	9.0%	8.2%	8.4%	8.1%	8.2%	8.2%	7.8%	8.3%	8.1%	8.3%
55-59 years	8.9%	8.3%	9.1%	8.3%	8.4%	8.0%	8.3%	8.0%	7.8%	8.3%	8.2%	8.4%
60-64 years	8.9%	8.3%	8.9%	8.2%	8.2%	8.0%	8.3%	8.1%	7.7%	8.4%	8.3%	8.8%
65-69 years	8.9%	8.5%	9.1%	8.2%	8.2%	7.8%	8.2%	8.1%	7.7%	8.3%	8.1%	8.8%
70-74 years	9.1%	8.7%	9.3%	8.3%	8.3%	7.8%	8.2%	7.9%	7.6%	8.2%	8.1%	8.6%
75-79 years	9.1%	8.6%	9.4%	8.3%	8.2%	7.7%	8.0%	7.8%	7.6%	8.2%	8.2%	8.9%
80-84 years	9.1%	8.7%	9.3%	8.3%	8.1%	7.6%	8.0%	7.8%	7.5%	8.3%	8.3%	9.1%
85-89 years	9.2%	8.8%	9.4%	8.3%	8.0%	7.5%	7.9%	7.8%	7.4%	8.3%	8.3%	9.2%
90-94 years	9.1%	8.9%	9.4%	8.1%	7.9%	7.4%	7.7%	7.6%	7.4%	8.5%	8.5%	9.5%
95+ years	9.7%	9.1%	9.8%	8.3%	7.8%	7.3%	7.6%	7.3%	7.3%	8.3%	8.2%	9.2%

Analogously, we define $f_{y,m}$ and obtain the results in Table 2.

**Table 2:** Estimated proportion of female deaths in month $m$ .

Age\month	1	2	3	4	5	6	7	8	9	10	11	12
0-14 years	8.8%	8.5%	9.1%	8.1%	8.0%	8.5%	8.0%	7.9%	8.1%	8.2%	7.9%	8.9%
15-29 years	8.7%	8.6%	8.8%	8.1%	8.2%	7.9%	8.5%	8.4%	8.3%	8.0%	7.9%	8.6%
30-34 years	8.5%	7.6%	9.1%	8.5%	8.1%	8.3%	7.9%	8.7%	8.2%	8.3%	7.9%	9.2%
35-39 years	8.4%	8.0%	8.5%	8.2%	8.6%	8.1%	8.4%	8.6%	8.2%	8.2%	8.0%	8.8%
40-44 years	9.1%	8.5%	9.2%	8.2%	8.3%	8.1%	8.1%	7.9%	7.9%	8.3%	8.0%	8.2%
45-49 years	9.0%	8.3%	9.0%	8.2%	8.3%	8.2%	8.2%	8.1%	7.8%	8.4%	8.1%	8.4%
50-54 years	8.8%	8.3%	8.9%	8.1%	8.3%	7.9%	8.2%	8.1%	8.0%	8.3%	8.5%	8.6%
55-59 years	8.9%	8.3%	8.8%	8.2%	8.3%	7.9%	8.2%	8.1%	7.9%	8.4%	8.3%	8.7%
60-64 years	8.9%	8.4%	9.1%	8.1%	8.3%	8.0%	8.2%	8.1%	7.7%	8.2%	8.2%	8.8%
65-69 years	9.0%	8.5%	9.2%	8.2%	8.2%	7.8%	8.2%	8.0%	7.7%	8.3%	8.1%	8.7%
70-74 years	9.1%	8.7%	9.3%	8.4%	8.2%	7.7%	8.1%	7.9%	7.7%	8.2%	8.0%	8.7%
75-79 years	9.1%	8.6%	9.4%	8.3%	8.1%	7.7%	8.0%	8.0%	7.6%	8.2%	8.2%	8.8%
80-84 years	9.1%	8.9%	9.5%	8.3%	8.0%	7.6%	8.0%	7.9%	7.5%	8.1%	8.2%	8.9%
85-89 years	9.4%	9.1%	9.8%	8.4%	8.0%	7.4%	7.9%	7.7%	7.4%	8.0%	8.0%	8.8%
90-94 years	9.2%	9.0%	9.7%	8.3%	7.9%	7.4%	7.9%	7.7%	7.4%	8.1%	8.2%	9.2%
95+ years	9.6%	9.3%	10.0%	8.3%	7.8%	7.2%	7.8%	7.6%	7.2%	8.1%	8.2%	9.0%

The mortality factors have been concentrated around their mean during the last years, with the empirical standard deviation being below 1.5% for all age groups, mainly around 0.5%.

Then, we distribute the expected number of deaths for year $t=2020,\, 2021,\, 2022$ according to the factors $f_{x,m}$ and $f_{y, m}$, \begin{equation} \mathbb E D_{x, t, m} = f_{x , m} \mathbb E D_{x, t} , \mathbb E D_{y, t, m} = f_{y , m} \mathbb E D_{y, t} , \end{equation} and set \begin{equation} \mathbb E D_{a, t, m} = \sum\limits_{x \in a} \mathbb E D_{ x, t, m} + \sum\limits_{ y \in a} \mathbb E D_{y, t, m} , \ \ m=1, \dots, 12, \end{equation} yielding the expected number of deaths in month $m$. We consider 2020 to be a leap year. The expected values should be compared to the observed data

\begin{equation}d_{a,t, m}=\sum\limits_{x \in a} d_{ x, t, m} + \sum\limits_{ y \in a} d_{y, t, m} , \ \ m=1, \dots, 12 . \end{equation}

Note that we do not assume that the population or the age structure is constant during a year. We just assume that the mean population change in the last years is comparable to the situation in 2020 to 2022, and thus, the changes in the last 10 years mimic the changes in 2020 to 2022.

## Results

Yearly expected mortality

Following the previously described method, we compute the expected number of deaths in 2020, 2021, and 2022. To compare the expected number of deaths $\mathbb E D_{a,t}$ in the age group $a$ to the observed values $d_{a ,t}$, we use the relative difference \begin{equation} \frac{d_{a,t}-\mathbb E D_{a,t}}{ \mathbb E D_{a,t}} . \end{equation} Table 3 gives the expected and observed number of deaths in the age groups \begin{equation} a\in\{0\text{-}14,15\text{-}29,30\text{-}39, \dots , 80\text{-}89, 90+ \}, \end{equation} as well as the absolute and relative differences.

**Table 3:** Expected deaths and yearly excess mortality for different age groups.

Age range (years)	*t *= 2020			*t *= 2021			*t *= 2022		
	Expected			Expected			Expected		
	Observed	Abs. diff.	Rel. diff.	Observed	Abs. diff.	Rel. diff.	Observed	Abs. diff.	Rel. diff.
0-14	3,531			3,513			3,517		
	3,306	-225	-6.38%	3,368	-145	-4.14%	3,580	63	1.79%
15-29	3,944			3,817			3,755		
	3,844	-100	-2.53%	3,934	117	3.07%	4,148	393	10.46%
30-39	6,626			6,585			6,546		
	6,668	42	0.64%	6,812	227	3.44%	7,182	636	9.72%
40-49	15,345			14,877			14,601		
	15,507	162	1.06%	16,095	1,218	8.19%	15,756	1,155	7.91%
50-59	58,641			57,705			56,471		
	57,331	-1,310	-2.23%	59,350	1,645	2.85%	56,777	306	0.54%
60-69	117,432			118,456			119,983		
	118,460	1,028	0.88%	126,781	8,325	7.03%	128,760	8,777	7.32%
70-79	198,389			190,335			186,303		
	201,957	3,568	1.80%	204,839	14,504	7.62%	206,108	19,805	10.63%
80-89	378,459			392,535			404,994		
	378,406	-53	-0.01%	398,041	5,506	1.40%	422,128	17,134	4.23%
90+	199,191			201,884			202,375		
	200,093	902	0.45%	204,467	2,583	1.28%	219,645	17,270	8.53%
Total	981,557			989,707			998,545		
	985,572	4,015	0.41%	1,023,687	33,980	3.43%	1,064,084	65,539	6.56%

The deviations of observed from the expected values must be compared to the deviation inherent to the parameter choice of our model and the empirical standard deviation that has occurred in the previous years. Overall, in 2020, the observed number of deaths was an increase of 0.4% extremely close to the expected number concerning the empirical standard deviation. By contrast, in 2021, the number of observed deaths was 3.4% higher than the number of expected deaths, which represents excess mortality of more than twice the empirical standard deviation. In 2022, the number of observed deaths was even 6.6% higher than the number of expected deaths which represents excess mortality of more than four times the empirical standard deviation.

**Figure 1:**
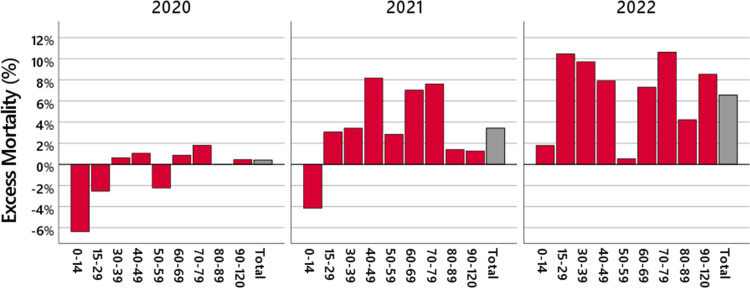
Yearly excess mortality. The red bars show the excess mortality in 2020 (left panel), 2021 (middle panel), and 2022 (right panel) in different age groups. The gray bars are the total excess mortality.

Figure 1 illustrates that the deviation of the observed mortality from the expected mortality is not uniform over the different age groups and that the pattern across the age groups changes from 2020 to 2021 and 2022.

The excess mortality observed in 2021 is almost entirely due to an increase in deaths in the age groups between 15 and 79 years. The highest values are reached in the age group 40-49 years, where the number of observed deaths was 8.2% higher than the number of expected deaths, which represents an excess mortality of more than five times the empirical standard deviation, and in the age groups 60-69 and 70-79 years, where the number of observed deaths was 7.0% and 7.6% higher than the number of expected deaths, which represents an excess mortality of more than twice the empirical standard deviation.

In 2022, the excess mortality is above 7% for nearly all age groups above 15 years. The highest values are reached in the age groups 15-29, 30-39, and 70-79 years, where an increase in the number of deaths is observed that is about 10% higher than expected. In the age groups 15-29, 30-39, and 60-89 years, an excess mortality of more than twice the empirical standard deviation is observed, and in the age groups 40-50 and 90+ years, an excess mortality of more than four times the empirical standard deviation is observed. The other age groups are below twice the empirical standard deviation.

An exception for all three years is the age group 50-59 years where, in contrast to the surrounding age groups, a substantially lower excess mortality is observed. This is also visible if the 2017/2019 life table by the Federal Statistical Office of Germany is replaced by a life table from another year, and several more detailed investigations from our side, as well as the investigations by De Nicola et al. [7,8], confirm this observation. We are not aware of an explanation for this fact. An interesting avenue for future research may be to explore what factors make this age group so resilient.

It should be pointed out that in the last 20 years, the maximal excess mortality in a year was about 25,000 deaths, and the authors are not aware of an excess mortality of more than 60,000 deaths - or in two consecutive years about 100,000 deaths - in the last decades.

Data uncertainty and model uncertainty

As already pointed out, first, there is an inherent data uncertainty of approximately 2,000-3,000 deaths. Second, to estimate the model uncertainty, we replace the 2017/2019 life table of the Federal Statistical Office of Germany with the life tables 2016/2018 or 2015/2017 and use different longevity factors, or ignore them. In Table 4, we present the total expected number of deaths $\mathbb E D_{t}$ and the excess mortality using the relative difference for different life tables and taking into account either none, half, or the full longevity trend.

**Table 4:** Expected deaths and excess mortality for different life tables.

Longevity trend	Life table	$\mathbb E D_{2020}$	Excess mort.	$\mathbb E D_{2021}$	Excess mort.	$\mathbb E D_{2022}$	Excess mort.
None	2015/2017	1,010,478	-2.46%	1,025,768	-0.20%	1,041,319	2.19%
None	2016/2018	999,583	-1.40%	1,014,802	0.88%	1,030,423	3.27%
None	2017/2019	988,288	-0.27%	1,003,270	2.04%	1,018,827	4.44%
Half	2015/2017	989,964	-0.44%	998,213	2.55%	1,006,620	5.71%
Half	2016/2018	986,013	-0.04%	994,294	2.96%	1,002,869	6.10%
Half	2017/2019	981,557	0.41%	989,707	3.43%	998,545	6.56%
Full	2015/2017	969,896	1.62%	971,451	5.38%	973,159	9.34%
Full	2016/2018	972,640	1.33%	974,230	5.08%	976,105	9.01%
Full	2017/2019	974,875	1.10%	976,341	4.85%	978,263	8.77%
	Observed	985,572		1,023,687		1,064,084	

The life tables have a significant effect on the question whether of excess mortality exists. The use of the life table 2015/2017 of the Federal Statistical Office of Germany without the longevity trend yields for the first two Corona years 2020 and 2021 even a mortality deficit. And when keeping half the longevity trend, in 2021, the excess mortality of 33,980 deaths for the life table 2017/2019 should be compared to the smaller excess mortality of 25,474 deaths when using the life table 2015/2017, the total difference being 8,506 deaths.

Hence, the life tables of the Federal Statistical Office of Germany have a serious fluctuation over the years, which should be taken into account as the model uncertainty. In the light of these results, we have decided to choose a model that avoids the extremes and includes half of the longevity factor. In this case, the range between the three models - which is an indicator of model uncertainty - is in all three years approximately 8,500 deaths per year.

**Figure 2:**
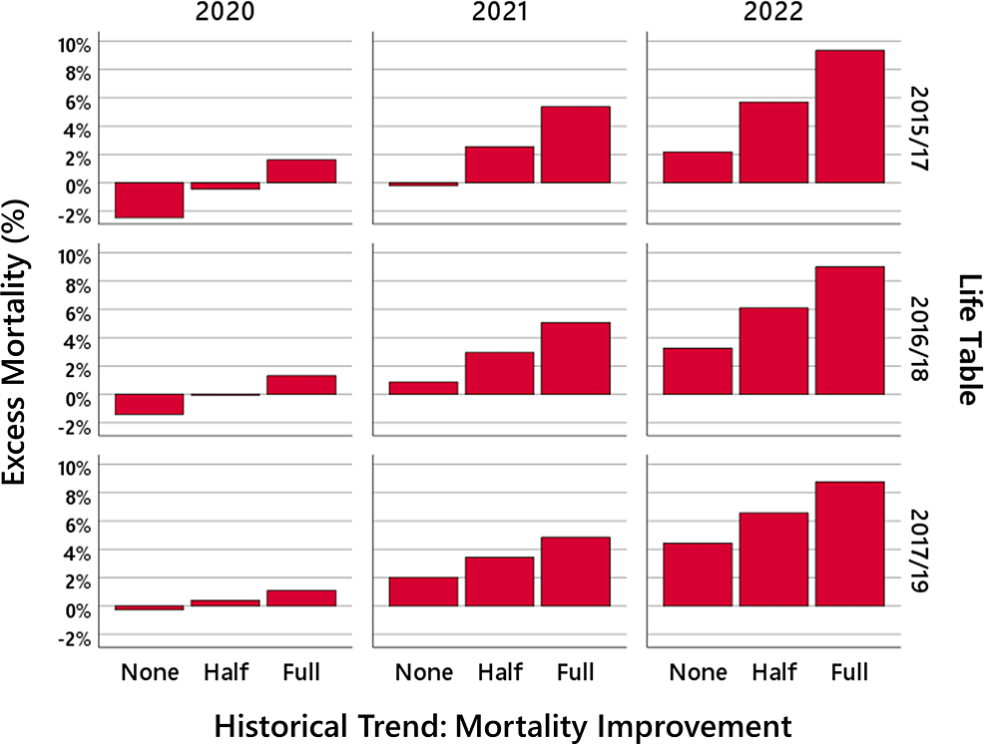
The model uncertainty. The bars show the mortality deficit and the excess mortality in 2020 (left panel), 2021 (middle panel), and 2022 (right panel) for different life tables and longevity trends.

In all these results obtained by life tables of recent years of the Federal Statistical Office of Germany, and in most other models [5-9], the main point coincides with our results: for 2020, the number of deaths is close to the expected value, whereas for 2021, there is noticeable excess mortality, and for 2022, there is a huge mortality excess, which has not been observed during the last decades. This is shown in Figure 2.

The empirical standard deviation

**Figure 3:**
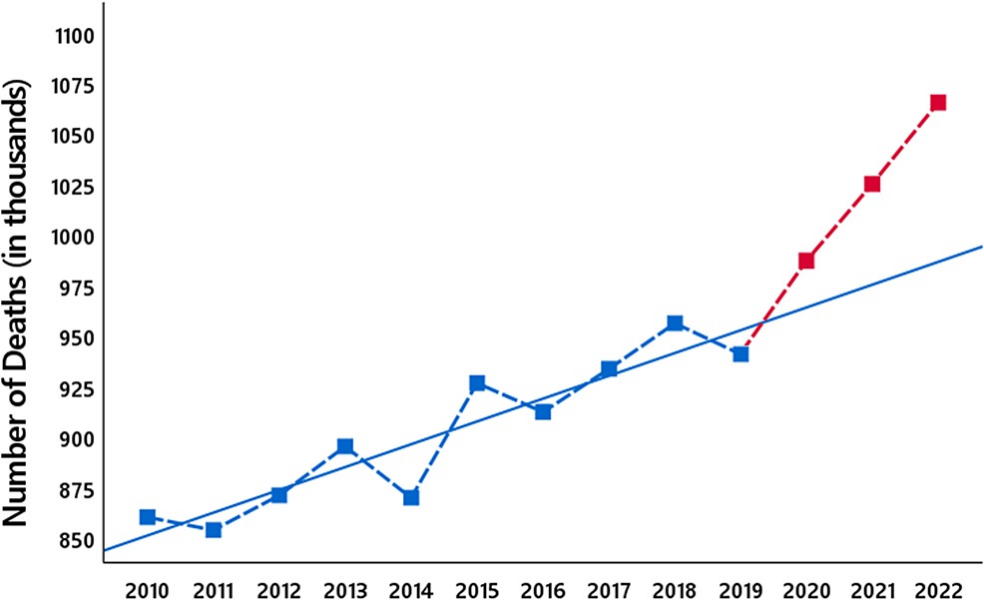
The empirical standard deviation. The blue squares show the number of all-cause deaths in Germany from 2010 to 2019, and the red squares the number of all-cause deaths in the years 2020 to 2022. The blue line shows the regression line for the years 2010 to 2019.

We use a linear regression model to approximate the empirical standard deviation $\hat \sigma (d_t)$ of the total number of deaths in year $t$ over all age groups. The regression leads to \begin{equation} d_{t} \approx L(t) = 837,711.9 + 11,336.2 \cdot (t-2009) \end{equation} for $t=2010,\dots,2019$ (Figure 3), which shows that each year, we expect a yearly increase of approximately 11,300 deaths in Germany. Observe that we have taken into account that 2012 and 2016 were leap years.

Calculating in this simple model the empirical standard deviation for 2010-2019 gives \begin{equation} \hat \sigma (d_{t}) = 14,162 . \end{equation} We do not claim that this is a precise estimate of the standard deviation $\sigma (D_t)$, yet we are convinced that this at least reflects the order of magnitude. To check whether this order of magnitude is plausible, we also computed the empirical standard deviation for the years 2000-2009, using again the linear regression model. For these years, the empirical standard deviation is approximately $12,600$, which is the same order as for the years 2010-2019.

At first sight, this empirical standard deviation seems to be in contrast to the model for $D_{x,t}$, where we assumed that the number of deaths follows a binomial distribution. This natural assumption would imply that the variance is smaller than the number of deaths, approximately one million, and hence, the standard deviation is smaller than 1,000. Thus, in actuarial science, further randomization is introduced, which keeps the expectation unchanged - and thus, our results in the previous sections are still valid - but increases the variance to the observed 14,000.

We compare the excess mortality of approximately 4,000 deaths in 2020, 34,000 deaths in 2021, and 65,000 in 2022 to the empirical standard deviation $\hat \sigma$. In 2020, this leads to \begin{equation} d_{2020} - \mathbb E D_{2020} \approx 0.28 \hat \sigma , \end{equation} the number of deaths in 2020 is very close to the expected number. For 2021, we have \begin{equation} d_{2021} - \mathbb E D_{2021} \approx 2.40 \hat \sigma \end{equation} and for 2022 \begin{equation} d_{2022} - \mathbb E D_{2022} \approx 4.62 \hat \sigma . \end{equation} In many applications, an observed deviation beyond twice the standard deviation is called significant because for normally distributed random variables, the 5% confidence interval leads to this bound. For a normal distributed random variable, a bound of 4.62 times the standard deviation (occurring in 2022) leads approximately to a 0.0004% confidence interval. (Recall that we avoid the use of the words confidence interval.) In addition, one should also have in mind the data uncertainty of 2,000 to 3,000 deaths and the model uncertainty of approximately 4,250 deaths.

The same method, a linear regression model, can be applied to age groups $a$. Table 5 states the observed empirical variance $\hat \sigma (d_{a,t})$.

**Table 5:** Empirical standard deviations for different age groups.

Age range (years)	Emp. standard deviation
0-14	158
15-29	148
30-39	245
40-49	237
50-59	868
60-69	3,646
70-79	6,101
80-89	7,770
90+	4,005
Total	14,162

Comparing these to the values in Table 3 shows that the excess mortality in 2021 is more than twice the empirical standard deviation in the age groups 40-49, 60-69, 70-79 years and more than twice the empirical standard deviation in *all *age groups except 0-14 and 50-59 in 2022, whereas in 2020, for all age groups, the excess deaths are close to the expected value compared to the empirical standard deviation.

Monthly expected mortality

Following the computations described in the previous section, we calculate the expected number of deaths $\mathbb E D_{a, 2021, m}$ for all months $m=1, \dots, 12$, in the years $t=2020, 2021, 2022$. To compare the expected and the observed values, we use the relative difference \begin{equation} \frac{d_{a,2021, m}-\mathbb E D_{a,2021,m}}{ \mathbb E D_{a,2021, m}}. \end{equation} The results are given in Table 6.

**Table 6:** Expected deaths and monthly excess mortality over all age groups.

	*t *= 2020			*t *= 2021			*t *= 2022		
	Expected	Observed	Rel. diff.	Expected	Observed	Rel. diff.	Expected	Observed	Rel. diff.
*m *= 1	89,441	84,980	-4.99%	90,492	106,803	18.02%	91,328	89,440	-2.07%
*m *= 2	88,627	80,030	-9.70%	86,593	82,191	-5.08%	87,400	82,809	-5.25%
*m *= 3	92,263	87,396	-5.28%	93,345	81,901	-12.26%	94,203	93,754	-0.48%
*m *= 4	81,088	83,830	3.38%	82,022	81,877	-0.18%	82,762	86,222	4.18%
*m *= 5	79,013	75,835	-4.02%	79,895	80,876	1.23%	80,592	81,815	1.52%
*m *= 6	74,508	72,159	-3.15%	75,331	76,836	2.00%	75,979	79,468	4.59%
*m *= 7	78,389	73,795	-5.86%	79,268	76,704	-3.24%	79,960	85,968	7.51%
*m *= 8	76,809	78,742	2.52%	77,661	76,402	-1.62%	78,334	86,507	10.43%
*m *= 9	73,745	74,243	0.68%	74,564	77,931	4.52%	75,208	80,850	7.50%
*m *= 10	80,294	79,781	-0.64%	81,209	85,080	4.77%	81,926	94,237	15.03%
*m *= 11	80,143	85,989	7.30%	81,061	93,915	15.86%	81,779	88,674	8.43%
*m *= 12	87,237	108,792	24.71%	88,266	103,171	16.89%	89,075	114,340	28.36%

These monthly mortality estimates reflect the excess deaths caused by the usual infections in winter and high-temperature weeks in summer. The excess mortality during the COVID-19 pandemic must be compared to these expected mortality waves.

**Figure 4:**
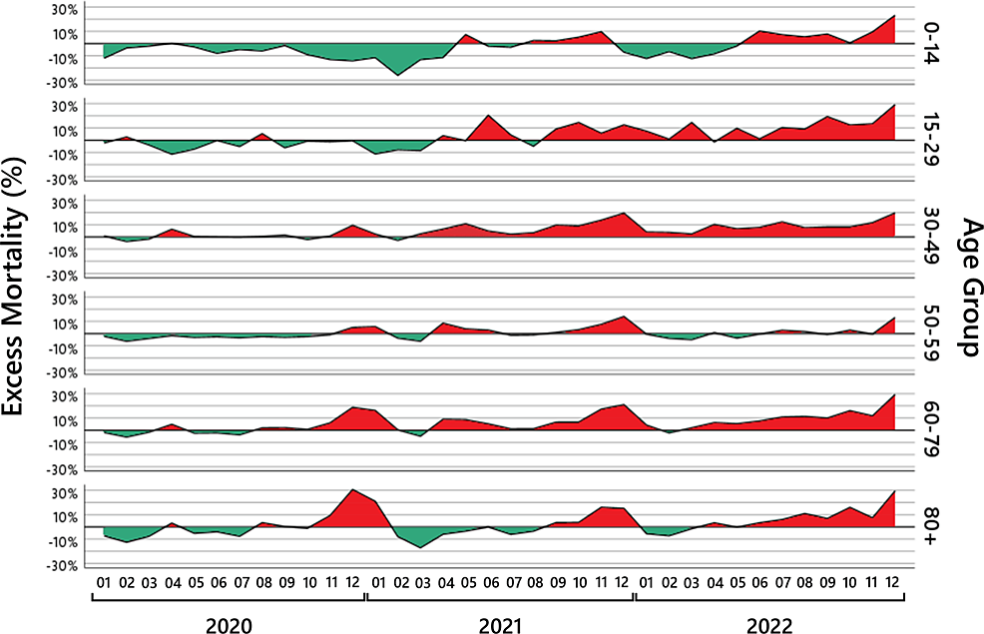
Monthly excess mortality. For six age groups, the black lines show the monthly excess mortality from January 2020 to December 2022. The red-shaded areas show the periods where a mortality increase was observed; the green-shaded areas show the periods where a mortality deficit was observed.

In the following sections, we investigate in detail the age ranges 0-14, 15-29, 30-49, 50-59, 60-79, 80+. Figure 4 shows the results for these age groups.

Children (0-14 years)

In the age group 0-14 years, the number of deaths is small and dominated by the relatively large infant mortality. The expected monthly number of deaths is approximately 300. In the binomial model - which, as we know, underestimates the standard deviation - we already expect oscillations at least of the order \begin{equation} 2 \sigma (D_{0-14, t ,m}) \geq 2\sqrt{D_{0-14, t ,m}} \approx 35 . \end{equation} Such deviations already lead to excess mortality of more than 10%. The results in Table 7 and the graph in Figure 4, age group 0-14 years, show, in fact, such abrupt oscillations; hence, we think that any conclusion relying on these numbers has to be taken with great care.

**Table 7:** Expected deaths and monthly excess mortality in the age group 0-14 years.

	*t *= 2020			*t *= 2021			*t *= 2022		
	Expected	Observed	Rel. diff.	Expected	Observed	Rel. diff.	Expected	Observed	Rel. diff.
*m *= 1	309	272	-11.96%	308	273	-11.45%	309	271	-12.19%
*m *= 2	302	291	-3.52%	291	215	-26.02%	291	272	-6.50%
*m *= 3	319	313	-2.01%	319	277	-13.10%	319	280	-12.26%
*m *= 4	288	289	0.24%	288	255	-11.37%	288	264	-8.34%
*m *= 5	285	277	-2.72%	284	305	7.34%	284	279	-1.92%
*m *= 6	299	275	-7.97%	298	292	-2.08%	299	329	10.21%
*m *= 7	292	278	-4.82%	291	283	-2.91%	292	313	7.27%
*m *= 8	290	273	-5.98%	290	297	2.49%	290	306	5.49%
*m *= 9	281	277	-1.55%	281	287	2.21%	281	303	7.79%
*m *= 10	286	260	-8.99%	285	300	5.23%	285	287	0.56%
*m *= 11	276	240	-13.02%	275	302	9.68%	276	302	9.56%
*m *= 12	304	261	-14.10%	303	282	-6.99%	304	374	23.22%

Maybe the only notable result is the well-accepted fact that children are extremely robust to SARS-CoV-2 infections, and the curve seems to be independent of the usual SARS-CoV-2 infection waves. Exceptions are the months of May and November 2021 and June and November 2022, with a visible positive mortality excess, and December 2022 with a serious mortality excess.

Young adults (15-29 years)

As for the age group 0-14 years, the number of expected and observed deaths in the age group 15-29 years is small. Hence, again, the observed excess mortality in Table 8 has to be interpreted with great care. The numbers until March 2021 are mostly negative and reflect the minimal number of deaths by the two COVID-19 waves in this age range.

**Table 8:** Expected deaths and monthly excess mortality in the age group 15-29 years.

	*t *= 2020			*t *= 2021			*t *= 2022		
	Expected	Observed	Rel. diff.	Expected	Observed	Rel. diff.	Expected	Observed	Rel. diff.
*m *= 1	336	329	-2.22%	327	290	-11.19%	321	345	7.39%
*m *= 2	322	330	2.63%	301	278	-7.73%	296	299	0.87%
*m *= 3	333	320	-3.88%	323	296	-8.39%	318	364	14.51%
*m *= 4	325	288	-11.37%	315	327	3.69%	310	306	-1.38%
*m *= 5	335	311	-7.18%	325	324	-0.36%	320	351	9.72%
*m *= 6	329	329	-0.12%	320	385	20.43%	315	318	1.10%
*m *= 7	353	335	-5.08%	342	357	4.23%	337	372	10.39%
*m *= 8	339	357	5.35%	329	313	-4.83%	324	353	9.09%
*m *= 9	325	305	-6.08%	315	344	9.15%	310	370	19.33%
*m *= 10	322	320	-0.65%	313	358	14.53%	308	346	12.51%
*m *= 11	313	309	-1.22%	304	321	5.74%	299	339	13.50%
*m *= 12	312	311	-0.33%	303	341	12.61%	298	385	29.23%

Somehow unexpectedly, in June 2021, a significant excess mortality is observed, followed by a decrease. However, other than at the beginning of the year, excess mortality remains above zero - with the exceptions of August 2021 and April 2022 - and has visible peaks in October and December 2021 and again in March and May 2022, and increases drastically in December 2022, reaching excess mortality of over 29%.

Adults (30-49 years)

The age group 30-49 years is the largest, and we expect approximately 1,800 deaths per month. The results are given in Table 9.

**Table 9:** Expected deaths and monthly excess mortality in the age group 30-49 years.

	*t *= 2020			*t *= 2021			*t *= 2022		
	Expected	Observed	Rel. diff.	Expected	Observed	Rel. diff.	Expected	Observed	Rel. diff.
*m *= 1	1,949	1,964	0.78%	1,908	1,952	2.29%	1,880	1,954	3.96%
*m *= 2	1,850	1,782	-3.69%	1,750	1,702	-2.75%	1,724	1,788	3.69%
*m *= 3	1,957	1,924	-1.68%	1,917	1,965	2.49%	1,889	1,935	2.42%
*m *= 4	1,816	1,929	6.25%	1,779	1,893	6.41%	1,753	1,932	10.22%
*m *= 5	1,841	1,845	0.24%	1,804	1,998	10.77%	1,778	1,896	6.67%
*m *= 6	1,789	1,788	-0.06%	1,753	1,837	4.82%	1,726	1,861	7.79%
*m *= 7	1,839	1,834	-0.29%	1,802	1,842	2.22%	1,776	1,994	12.30%
*m *= 8	1,814	1,821	0.38%	1,778	1,838	3.39%	1,752	1,884	7.55%
*m *= 9	1,736	1,762	1.48%	1,701	1,865	9.61%	1,677	1,814	8.19%
*m *= 10	1,804	1,766	-2.12%	1,768	1,927	9.02%	1,742	1,884	8.18%
*m *= 11	1,744	1,756	0.67%	1,709	1,944	13.75%	1,684	1,881	11.70%
*m *= 12	1,831	2,004	9.46%	1,794	2,144	19.52%	1,768	2,115	19.66%

As in the age group 15 to 29 years, the numbers in the year 2020 are mostly unremarkable and reflect the minimal number of deaths by the first COVID-19 wave in April 2020, and visible excess mortality in December 2020 in this age range. Then, the excess mortality fluctuates around zero until March 2021. From an actuarial perspective, we would expect this to continue until winter.

Somehow unexpectedly, in April and mainly in May 2021, a significant increase in excess mortality is observed, occurring one month before the similar excess mortality in the age group 15 to 29 years. The excess mortality in May is followed by a decrease up to August. However, other than at the beginning of the year, excess mortality remains above zero so the increase in excess mortality in April and May is not compensated for. In September, there is again significant excess mortality, which increases in November and reaches 20% in December 2021. In 2022, the excess mortality stays always positive, fluctuating around 8%, and reaches again serious excess mortality of nearly 20% in December.

The exceptional age group (50-59 years)

The age group 50-59 years seems to be exceptionally resilient against the factors that drive excess mortality in the other age groups. As can be seen in Table 10, there are neither huge peaks of mortality excess nor serious mortality deficits, the mortality excess fluctuates around zero. The numbers in the year 2020 are close to zero, ignoring the first COVID-19 wave in April 2020, and show some mild excess mortality in winter 2020 in this age range. There is a visible peak in April 2021 and December 2021. In 2022, the excess mortality is always close to zero, only in December, there is some serious excess mortality.

**Table 10:** Expected deaths and monthly excess mortality in the age group 50-59 years.

	*t *= 2020			*t *= 2021			*t *= 2022		
	Expected	Observed	Rel. diff.	Expected	Observed	Rel. diff.	Expected	Observed	Rel. diff.
*m *= 1	5,215	5,102	-2.18%	5,147	5,438	5.65%	5,037	5,013	-0.47%
*m *= 2	5,011	4,699	-6.23%	4,776	4,600	-3.68%	4,674	4,494	-3.85%
*m *= 3	5,242	5,036	-3.94%	5,174	4,853	-6.20%	5,063	4,814	-4.92%
*m *= 4	4,822	4,743	-1.65%	4,760	5,166	8.53%	4,659	4,695	0.78%
*m *= 5	4,894	4,742	-3.10%	4,830	5,014	3.82%	4,726	4,554	-3.65%
*m *= 6	4,666	4,547	-2.56%	4,605	4,736	2.85%	4,505	4,487	-0.41%
*m *= 7	4,810	4,647	-3.39%	4,748	4,686	-1.30%	4,647	4,772	2.70%
*m *= 8	4,743	4,629	-2.40%	4,680	4,631	-1.05%	4,579	4,656	1.68%
*m *= 9	4,599	4,461	-2.99%	4,538	4,582	0.96%	4,441	4,406	-0.79%
*m *= 10	4,873	4,755	-2.42%	4,809	4,962	3.17%	4,707	4,839	2.81%
*m *= 11	4,798	4,753	-0.94%	4,735	5,094	7.57%	4,634	4,615	-0.41%
*m *= 12	4,967	5,217	5.03%	4,903	5,588	13.97%	4,799	5,432	13.19%

This leads to the surprising result that in all pandemic years 2020 to 2022, this age group has - in contrast to all neighboring age groups - no notable excess mortality.

The retirement age (60-79 years)

This group consists of the ages 60 to 79 years, a mixed group where parts of this population are still healthy and parts are already vulnerable, and for these, a SARS-CoV-2 infection can be dangerous. The results in Table 11 confirm that there are serious fluctuations.

**Table 11:** Expected deaths and monthly excess mortality in the age group 60-79 years.

	*t *= 2020			*t *= 2021			*t *= 2022		
	Expected	Observed	Rel. diff.	Expected	Observed	Rel. diff.	Expected	Observed	Rel. diff.
*m *= 1	28,409	27,905	-1.77%	27,857	32,372	16.21%	27,627	28,787	4.20%
*m *= 2	27,910	26,369	-5.52%	26,423	26,505	0.31%	26,205	25,647	-2.13%
*m *= 3	29,147	28,708	-1.51%	28,569	27,195	-4.81%	28,326	28,896	2.01%
*m *= 4	26,058	27,314	4.82%	25,555	27,839	8.94%	25,347	26,933	6.26%
*m *= 5	25,811	25,201	-2.36%	25,320	27,507	8.64%	25,119	26,467	5.37%
*m *= 6	24,481	23,960	-2.13%	24,022	25,274	5.21%	23,836	25,637	7.56%
*m *= 7	25,625	24,683	-3.68%	25,148	25,440	1.16%	24,954	27,662	10.85%
*m *= 8	25,105	25,595	1.95%	24,631	24,939	1.25%	24,437	27,211	11.35%
*m *= 9	24,060	24,568	2.11%	23,603	25,164	6.62%	23,415	25,758	10.00%
*m *= 10	25,923	26,101	0.69%	25,422	27,119	6.67%	25,216	29,256	16.02%
*m *= 11	25,669	27,211	6.01%	25,164	29,519	17.31%	24,954	27,897	11.79%
*m *= 12	27,623	32,802	18.75%	27,076	32,747	20.95%	26,848	34,717	29.31%

This is visible in the results for 2020. A decent peak in April 2020 is followed by a significant peak around December 2020. The peak of December 2020 continues in January 2021 but then turns into a mortality deficit. In April 2021, we observe serious excess mortality for two months. In September and October 2021, we see a decent, and in November and December 2021, again significant excess mortality. The year 2022 starts with an unremarkable mortality deficit, which again in April turns into excess mortality, which remains for the rest of the year at a high level and is even above 29% in December.

Old ages (80+ years)

The last group consists of the ages $\geq 80$ (beyond the expected lifetime in Germany, which is approximately at the age of 80), where large parts of the vulnerable population belong, and a SARS-CoV-2 infection is particularly dangerous. The results in Table 12 for this age group show a decent peak for April 2020 and a huge peak around December 2020. The peak of December 2020 continues in January 2021 and then turns into a mortality deficit until April 2021, when the downward trend stops. In September and October, we see a decent, and in November and December 2021, serious excess mortality. The year 2022 starts with a mortality deficit, which again in June turns into excess mortality, which remains on a high level for the rest of the year and reaches an extremum in December, with more than 29% excess mortality.

**Table 12:** Expected deaths and monthly excess mortality in the age group (80+ years).

	*t *= 2020			*t *= 2021			*t *= 2022		
	Expected	Observed	Rel. diff.	Expected	Observed	Rel. diff.	Expected	Observed	Rel. diff.
*m *= 1	53,222	49,408	-7.17%	54,945	66,478	20.99%	56,154	53,070	-5.49%
*m *= 2	53,232	46,559	-12.54%	53,052	48,891	-7.84%	54,210	50,309	-7.20%
*m *= 3	55,264	51,095	-7.54%	57,043	47,315	-17.05%	58,287	57,465	-1.41%
*m *= 4	47,779	49,267	3.12%	49,324	46,397	-5.93%	50,404	52,092	3.35%
*m *= 5	45,848	43,459	-5.21%	47,332	45,728	-3.39%	48,365	48,268	-0.20%
*m *= 6	42,944	41,260	-3.92%	44,334	44,312	-0.05%	45,299	46,836	3.39%
*m *= 7	45,469	42,018	-7.59%	46,936	44,096	-6.05%	47,955	50,855	6.05%
*m *= 8	44,518	46,067	3.48%	45,954	44,384	-3.42%	46,952	52,097	10.96%
*m *= 9	42,744	42,870	0.30%	44,125	45,689	3.54%	45,083	48,199	6.91%
*m *= 10	47,087	46,579	-1.08%	48,612	50,414	3.71%	49,669	57,625	16.02%
*m *= 11	47,343	51,720	9.25%	48,873	56,735	16.09%	49,932	53,640	7.43%
*m *= 12	52,200	68,197	30.65%	53,887	62,069	15.18%	55,059	71,317	29.53%

Although the trend in the age groups 60 to 79 and 80+ years looks parallel, it is interesting to point out the differences. As can be seen in Figure 4, the curve for the age group 80+ years is below and somehow parallel to the curve for the age group 60 to 79 years. The main difference is the deviation of the age group 60 to 79 years in April and May 2021 where a jump in the mortality behavior for this age group is visible. The age group 80+ years seems to be more resistant to mortality causes at a larger scale than other age groups. At certain moments, some people die some months before or after the *expected* time of death, but the curve for excess mortality mostly oscillates around the 0% axes. A visible mortality deficit until October 2020 with exceptions in April and August is followed by a huge mortality excess peak at the turn of the year 2020/2021. This, in turn, is more or less compensated by the mortality deficit from January to July 2021, the peak around November and December 2021 is nearly compensated in February to March 2022. Something surprisingly, since August 2022, the mortality excess stays continuously on a very high level.

**Figure 5:**
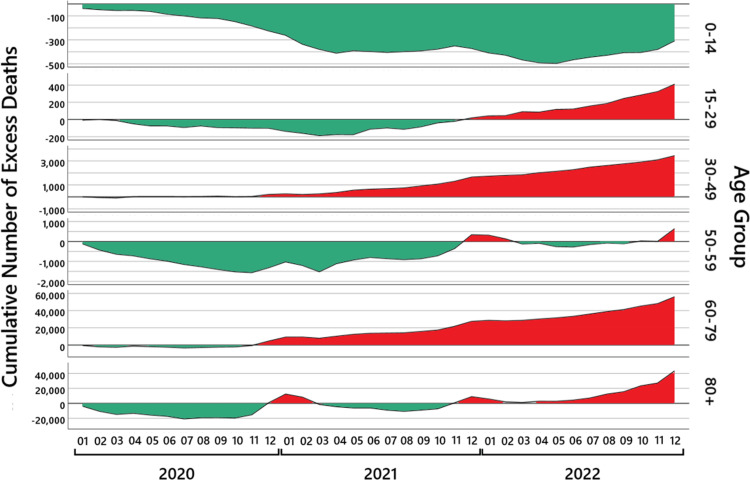
The cumulative excess mortality. For six age groups, the black lines show the cumulative number of excess deaths from January 2020 to December 2022. The green areas show the regions of a cumulative mortality deficit and the red areas of a cumulative excess mortality. Note that the $y$-axis of the number of cumulative excess deaths is scaled differently depending on the age group.

We make this observation visible by calculating the cumulative excess mortality in absolute numbers, which are shown in Figure 5. Maybe due to a comparably mild flu season in 2019/2020, the age group 80+ starts with a negative value. In July 2020, up to 20,000 people, more than expected, are still alive, which is compensated in December 2020 to February 2021, where the curve is 10,000 above the expectation, and then the curve fluctuates to -10,000, to +10,000, and until July 2022 where it is approximately 7,000. This shows that a mortality deficit or excess mortality in the age group $[80, \infty)$ usually just shifts the time of death by some months. This changes in the last months of 2022 where we see a cumulated excess mortality of 43,000 deaths at the end of the year. This is in contrast to the situation for the age group 60 to 79 years. The cumulative excess mortality is steadily increasing up to 56,000 deaths at the end of year 2022.

The children in the age group 0 to 14 years and the exceptional age group 50 to 59 years seem to be resilient, and the cumulative excess mortality is mostly negative. The age groups 15 to 29 and 30 to 49 years behave similarly to the age group 60 to 79 years, yet with the difference that the age group 15 to 29 years starts in summer 2021 from a mortality deficit and with a delayed increase in mortality of one to two months.

Stillbirths in the years 2019 to 2022 in Germany

In all previous studies on excess mortality during the COVID-19 pandemic, only the increase in the number of deaths of living persons has been examined. In the following, it is examined whether similar increases in mortality to that found for living persons are also found at the level of stillborn children.

One problem with analyzing excess mortality at the level of stillbirths in Germany is that the definition of *stillbirth* has been changed at the end of 2018. Up to this point, a stillborn child was considered a stillbirth if a birth weight of at least 500 g was reached. Since the end of 2018, a stillborn child is considered a stillbirth if at least 500 g or the 24th week of pregnancy was reached, which led to a diagnostically related increase in stillbirths. This means that the figures on stillbirths are only validly comparable from 2019 onward. Thus, estimating excess mortality at the level of stillbirths based on a modeling of long-term trends in mortality is problematic. Furthermore, the empirical standard deviation that occurred in the years before cannot be determined. Thus, we only descriptively report the course of stillbirths from 2019 onward.

**Figure 6:**
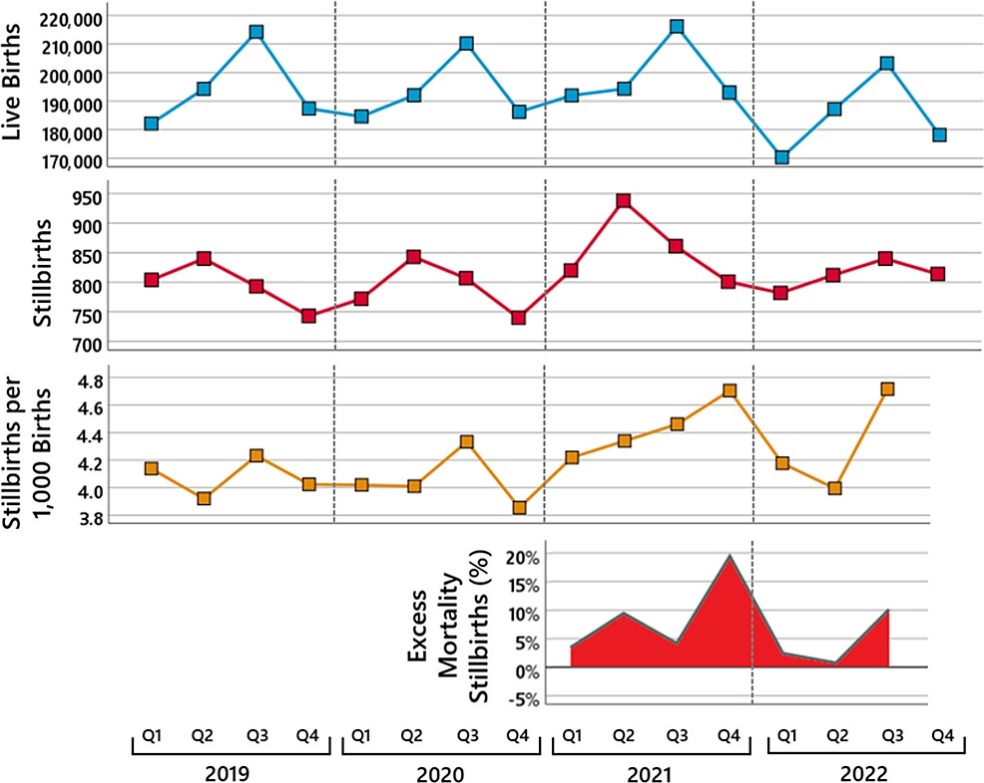
Stillbirths in the years 2019 to 2022 in Germany. The first panel shows the number of live births per quarter from 2019 to 2022, the second panel the number of stillbirths per quarter from 2019 to 2022, the third panel the number of stillbirths per 1,000 total births (sum of the number of stillbirths in a quarter and the number of live births in the following quarter) per quarter from 2019 to 2022, and the fourth panel the quarterly increase in the number of stillbirths per 1,000 total births in the years 2021 and 2022 compared to the mean across the years 2019 and 2020.

Note that the number of stillbirths must be interpreted in relation to the number of total births because an increase or decrease in the number of total births is automatically accompanied by an increase or decrease in stillbirths. Figure 6 shows in the first panel the number of live births per quarter [22] and in the second panel the number of stillbirths per quarter [23] since 2019. As can be seen from the shift in the seasonal peaks of the stillbirths compared to the seasonal peaks of the live births, stillbirths precede live births from the same pregnancy cohort by about one trimester. Thus, to correctly control for the effect of a general increase or decrease in the number of total births, the number of total births must be calculated as the sum of the number of stillbirths in a quarter and the number of live births in the following quarter.

Figure 6 (third panel) shows the number of stillbirths per 1,000 total births and the fourth panel shows the quarterly increase in the number of stillbirths per 1,000 total births in the years 2021 and 2022 compared to the mean across the years 2019 and 2020. Note that the number of stillbirths per 1,000 total births cannot be determined for the fourth quarter of 2022 because the number of live births in the first quarter of 2023 has not yet been published by the Federal Statistical Office of Germany.

Until the end of 2021, the number of live births shows a stable course with a regularly repeating seasonal pattern. In the first quarter of 2022, a sudden and sustained drop in the number of births is observed. Regarding the number of stillbirths, a stable course is observed until the end of the first quarter of 2021. In the second quarter of 2021, a sudden increase in stillbirths is observed, despite the stable course of live births until the end of 2021. Compared to the quarterly number of stillbirths per 1,000 total births in the years 2019 and 2020, the number of stillbirths increased by 9.4% in the second quarter of 2021 and by 19.4% in the fourth quarter of 2021. In 2022, the stillbirth rate stays unusually high, reaching a maximum in the third quarter. Note, however, that the quarterly pattern observed in the year 2022 must be interpreted with caution because only preliminary data is available based on the reporting month, with the data being assigned to the month of death only with the publishing of the final data by the Federal Statistical Office of Germany.

The observed increase in stillbirths is similar to the increases in mortality observed for living persons: in the year 2020, no change in stillbirths is found compared to the previous year; in the year 2021, a sudden increase in stillbirths is observed in the second quarter, which reaches a high level in the fourth quarter of 2021. A comparison with the empirical standard deviation occurring in the years before the change in the definition of stillbirths suggests that the observed increase in stillbirths represents a substantial increase. In the years 2007 to 2018, the quarterly stillbirths excess mortality (i.e., the increase/decrease in the rate of stillbirths per 1,000 total births compared to the mean across the two previous years) showed an empirical standard deviation of 4.9%. For instance, in the fourth quarter of 2021, an increase in stillbirths of about four standard deviations was observed.

## Discussion

In this study, we estimated the expected number of all-cause deaths and the increase in all-cause mortality for the pandemic years 2020 to 2022 in Germany. The results revealed several previously unknown mortality dynamics that require a reassessment of the mortality burden brought about by the COVID-19 pandemic.

The analysis of the yearly excess mortality showed a marked difference between the pandemic years 2020, 2021, and 2022. Cumulated over all age ranges and months, in the year 2020, the observed number of deaths was close to the expected number; yet, in 2021, the observed number of deaths was far above the expected number (with an excess mortality of 34,000 deaths, more than twice the empirical standard deviation), and further increased in 2022 (with an excess mortality of 66,000 deaths, above four times the standard deviation). An age-dependent analysis showed that the strong excess mortality observed in 2021 and 2022 was mainly due to an above-average increase in deaths in the age groups between 15 and 79 years. The analysis of the monthly excess mortality in the age groups between 15 and 79 years showed that the high excess mortality started to accumulate from April 2021 onward. A similar pattern was observed for the number of stillbirths, which was similar to the previous years until March 2021, after which also a sudden and sustained increase was observed.

The findings of this study are in line with previous studies that have examined excess mortality in the years 2020 and 2021 based on estimation methods that take into account changes in population sizes [7-10,13]. In all of these studies, the estimated excess mortality was much higher in 2021 than in 2020 where the excess mortality estimations showed no substantial increase or even a decrease in mortality. For instance, in the recent study by Levitt et al. [13] where excess mortality was estimated based on a multiverse analysis approach, it was estimated that mortality decreased in Germany in 2020 by 0.1% and increased in 2021 by 2.4%. According to the mortality estimations for the years 2020 and 2021 in the two studies by De Nicola et al. [7,8] where similar estimation methods were used than in this study, mortality increased in 2020 in Germany by 0.6% and in 2021 by 2.3%.

The excess mortality estimates for the years 2020 and 2021 reported in this study are highly similar to the estimations reported in these previous studies, which demonstrates the validity of our estimates. In particular, in line with this study, in the two studies by De Nicola et al. [7,8], a similar shift in excess mortality from older to younger age groups is reported from 2020 to 2021. While in 2020, excess mortality was most pronounced in the oldest age group of 90+, in 2021, excess mortality was most pronounced in the middle age groups. Going beyond the previous studies, this study demonstrates that excess mortality shows another sharp increase in 2022 across all age groups down to the youngest age groups.

Possible factors influencing mortality

The findings of this study raise the question of what happened in spring 2021 that led to a sudden and sustained increase in mortality, although no such effects on mortality had been observed during the early COVID pandemic so far. In the following sections, possible explanatory factors are explored.

The number of deaths in a year depends on several different factors, the most important being maybe the severity of the flu and the number of extremely hot weeks. The fluctuations between different years, and thus, the approximation of the empirical standard deviation $\hat \sigma (D_t)$, include all these factors. It is rather subjective, and most probably impossible, to precisely define *extreme events*, calculate the influence of such extreme events, and adjust mortality to entirely normal years. Thus, our calculations give the expected number of deaths, taking into account all these extreme and non-extreme effects. We tried to quantify the sensitivity of our approach in the previous sections against the background of extreme events in the last years.

For the pandemic years 2020 to 2022, it is clear that the number of deaths has been influenced directly and indirectly by COVID-19. First, there has been a serious number of COVID-19 deaths, either as the only reason for death or in combination with several other causes, which also might have caused death independently of COVID-19. Second, the vaccination campaign that started in 2021 should be visible in reduced excess mortality or even better as a mortality deficit. An attempt to compare our results to the reported number of COVID-19 deaths and the number of vaccinations is the content of the next sections.

Third, the indirect effects on mortality due to the COVID-19 measures are extremely harder to quantify. Several aspects may contribute to excess mortality or a mortality deficit. In Germany, strict control measures since 2020 limited personal freedom, schools were partially closed, and there were severe lockdowns. This substantially influenced the risk of road accidents [24] and other outdoor casualties. On the other hand, many clinical services have been delayed or avoided in 2020, 2021, and 2022 [25]. All these and many more factors influenced mortality in different directions and on different time scales, but most of them are hard to measure, are highly correlated. It seems to be impossible to quantify the overall impact of the control measures on the number of deaths.

COVID-19 deaths and mortality

In this section, we compare the excess mortality since March 2020 to the reported number of COVID-19 deaths by the German Robert Koch Institute. The Robert Koch Institute provides the weekly number of COVID-19 deaths [26] for the age groups 0-9, 10-19, and so on; which differ from the age groups used by the Federal Statistical Office of Germany; in addition, these numbers are incomplete because all numbers below four are not stated due to data security reasons.

**Figure 7:**
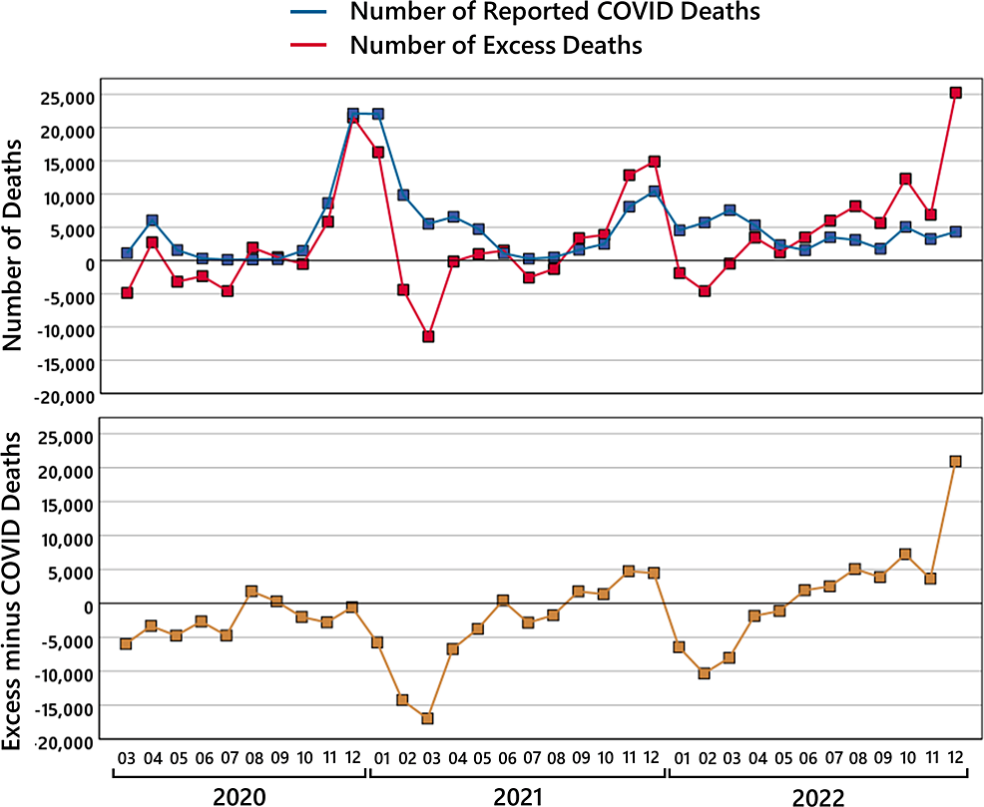
COVID-19 deaths versus excess mortality. The blue squares show the number of reported COVID-19 deaths, the red squares the mortality deficit and the excess mortality, and the yellow squares the difference between the number of excess deaths and the number of COVID-19 from March 2020 to December 2022.

Even when the reporting system in Germany seems to be partially insufficient, there should be a serious correlation between the reported number of deaths and excess mortality. To make the difference between the excess deaths and the COVID-19 deaths visible, we show the monthly development of the number of reported COVID-19 deaths and the excess mortality in the upper panel and on the same scale the difference between both in the lower panel of Figure 7.

Until July 2020, the number of excess deaths is below the number of reported COVID-19 deaths, and except for April 2020, a mortality deficit is observed despite the reporting of COVID-19 deaths. From August 2020 to December 2020, the numbers of excess deaths and reported COVID-19 deaths largely coincide. However, after that, the number of COVID-19 deaths stays at a high level while all-cause mortality decreases, and in February and March 2021, a noticeable all-cause mortality deficit is observed despite a high number of reported COVID-19 deaths of up to $10,000$. Starting in September 2021, a marked increase in excess mortality is observed that is not accompanied by a comparable increase in reported COVID-19 deaths. From January 2022 onward, both curves decouple, and from June 2022 onward, the number of excess deaths is increasingly larger than the number of reported COVID-19 deaths: in December 2022, nearly $25,000$ excess deaths are observed but only $4,330$ COVID-19 deaths reported. It is, thus, obvious that the number of reported COVID-19 deaths is fluctuating somehow independently of the excess mortality and contains a large number of expected deaths.

Because the Robert Koch Institute uses different age groups than the Federal Statistical Office of Germany, we divide the number of COVID-19 deaths in the age group 10-19 years into two equal parts to obtain the number of COVID-19 deaths in the age groups 0-14 and 15-29 years, estimate the number of deaths for those weeks with less than four deaths, and divide each week where two months overlap between these two months.

In Table 13, we list the number of excess deaths in six age groups and compare these to the approximated COVID-19 deaths, as a timetable we use the first pandemic year April 2020 to March 2021 and compare this to the second year April 2021 to March 2022 and to the recent months April 2022 to December 2022.

**Table 13:** Expected vs. observed deaths and excess vs. COVID-19 deaths.

Age range (years)	April 2020 to March 2021		April 2021 to March 2022		April 2022 to December 2022	
	Expected		Expected		Expected	
	Observed	Abs. diff.	Observed	Abs. diff.	Observed	Abs. diff.
		COVID		COVID		COVID
0-14	3,519		3,514		2,599	
	3,195	-324	3,426	-88	2,757	158
		15		55		37
15-29	3,904		3,801		2,820	
	3,729	-175	4,078	277	3,140	320
		61		102		45
30-49	21,790		21,380		15,654	
	22,124	334	22,965	1,585	17,261	1,607
		589		1,254		300
50-59	58,269		57,383		41,697	
	57,385	-884	58,780	1,397	42,456	759
		2,092		3,075		835
60-79	313,204		308,100		224,127	
	323,507	10,303	328,878	20,778	251,538	27,411
		21,399		18,680		8,426
80+	580,971		598,030		438,718	
	594,121	13,150	600,668	2,638	480,929	42,211
		54,012		30,568		20,585
total	981,656		992,209		725,615	
	1,004,061	22,405	1,018,795	26,586	798,081	72,466
		78,168		53,734		30,228

In the first two pandemic years, the number of reported COVID-19 deaths mostly exceeds the excess deaths. This changes in the last months when the number of reported COVID-19 deaths is decreasing, yet excess mortality is heavily increasing (apart from the exceptional group 50-59 years). It seems difficult to find a convincing pattern that explains the dependence of the excess deaths on COVID-19 deaths.

Beyond the problem that the number of reported COVID-19 deaths cannot be validly used to assess the effects of the COVID-19 pandemic on mortality, it seems also unlikely that the high excess mortality in 2021 in the age groups under 80 years can be explained by COVID-19 deaths because the marked increases in excess mortality in April to June 2021 - the mortality increases abruptly by 13% from March to April 2021 in the age group between 15 and 60 years - and also in October to December 2021 were not accompanied by comparable increased in the number of COVID-19 deaths. Furthermore, it seems also very unlikely that the abrupt increase in mortality in spring 2021 is due to delayed or avoided clinical services, which should lead to much smoother changes or due to side effects of COVID-19 measures. This is more unlikely in the year 2022 when excess mortality increases even further despite a decrease in reported COVID-19 deaths and although clinical care should slowly return to normal. It remains to investigate the factors that could have led to the surprising increase in excess mortality in spring 2021, fall 2021, and year 2022.

Taken together, it is misleading to measure the risk of the COVID-19 pandemic only using the reported COVID-19 deaths. One should rather use the excess mortality curve than the number of reported COVID-19 deaths, or a combination of both, to carve out the moments of high risk and to evaluate the total risk of a pandemic.

COVID-19 vaccination and mortality

In April 2021, an extensive COVID-19 vaccination campaign started in Germany. Regarding the relationship between excess mortality and vaccinations, if the vaccinations successfully prevent people from dying from COVID-19, a straightforward prediction is that excess mortality should decrease with an increased number of vaccinated persons.

**Figure 8:**
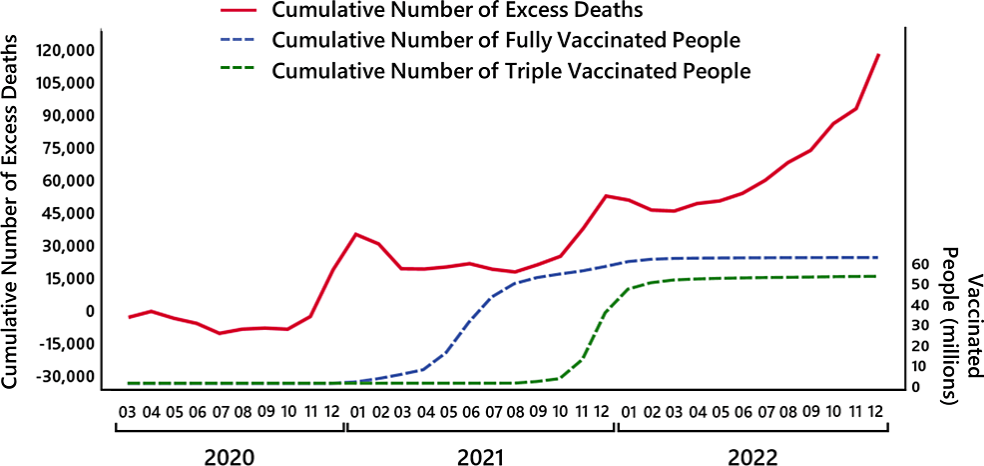
Number of vaccinations versus excess mortality. Cumulative number excess of deaths (red line) and a cumulative number of fully vaccinated (blue dashed line) and triple vaccinated (green dashed line) people from March 2020 to December 2022.

To explore this hypothesis, the course of the cumulative number of fully vaccinated and triple vaccinated persons recorded by the Robert Koch Institute [27] and the cumulative number of excess deaths since the beginning of the pandemic is shown in Figure 8. At times, when more people are dying than expected, the cumulative number of excess deaths increases, and when less people are dying than expected, the cumulative number of excess deaths decreases.

**Figure 9:**
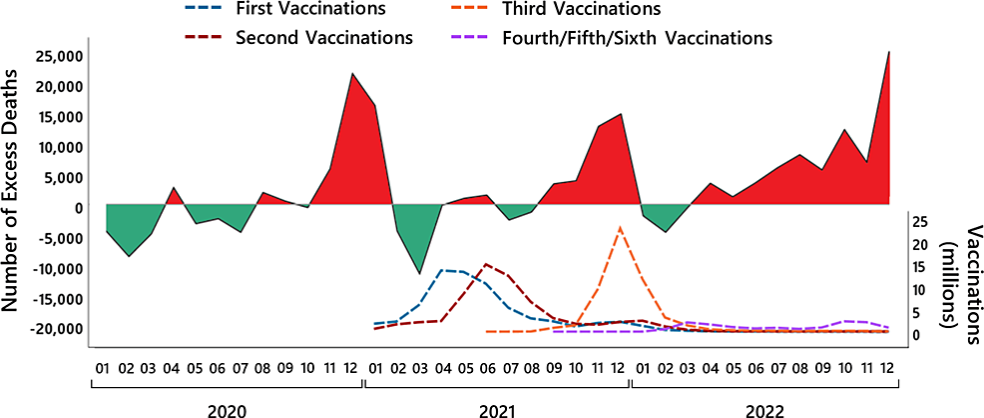
Number of vaccinations versus excess mortality. The red line shows the death deficit and the excess deaths, and the four dashed lines the number of vaccinations from January 2021 to December 2022.

As is visible in Figure 8, the obvious hypothesis of a decrease in excess mortality with an increasing number of vaccinated persons is not correct. During periods when many persons were vaccinated, excess mortality seems to have increased more strongly compared to the same periods in the previous pandemic year. During the first and second vaccination periods in spring and summer 2021, an increase in cumulative excess mortality is observed, while the year before a decrease was observed. During the period of the third vaccination, parallel to the increase in vaccinations, an increase in cumulative excess mortality can be observed that starts earlier than in the year before. And in 2022, when large parts of the population have been vaccinated, the cumulative number of excess deaths showed a further increase, which even exceeds the previous pandemic year without vaccinations. There seem to be negative long-term effects either of the SARS-CoV-2 infections, COVID-19 measures, the COVID-19 vaccination, or most probably a combination of these. To further explore the short-term relationship between vaccinations and excess mortality, the courses of the number of vaccinated persons and the number of excess deaths per month are shown in Figure 9.

Inspecting the numbers of vaccinations and excess deaths per month confirms the above impression: Other than in the year before, during the months with a high number of the first, second, and third vaccinations, also a high number of excess deaths was observed. The temporal relationship between the courses of vaccinations and excess deaths is especially pronounced for the third vaccination. In September and October 2021, the initial small increase in the number of third vaccinations was accompanied by a comparably small increase in excess deaths. In November and December 2021, the number of third vaccinations increased sharply, accompanied by a comparably sharp increase in excess deaths. In January 2022, the number of third vaccinations decreased sharply, accompanied by a comparably sharp decrease in the number of excess deaths.

Taken together, in 2021, with the beginning of the COVID-19 vaccination campaign, a higher excess mortality is observed than in the previous year in the months when large numbers of persons were vaccinated. In 2022, when large parts of the population were fully or even triple vaccinated, excess mortality is constantly increasing from spring onward, reaching a maximum of 28% in December. Such an observation is difficult to reconcile with the assumption that COVID-19 vaccinations are highly effective against COVID-19 deaths. Either the vaccinations are not as successful as expected, or the vaccinations successfully prevent COVID-19 deaths, but there are suddenly other factors than COVID-19 that lead to an increasing number of unexpected deaths in 2021 and 2022.

Regarding the latter possibility, it is interesting to consider the course of excess mortality in the different age groups shown in Figure 4. The mortality wave at the turn of the year from 2020 to 2021 is characterized by a strong age dependency, which follows the age-dependent risk of COVID-19: excess mortality is higher in older people and is completely absent in the age groups below 30 years. This pattern is substantially changing from April 2021 onward at the time when more and more people were vaccinated. Suddenly, excess mortality appears that is no longer dependent on age and even observed in young age groups. This speaks against the possibility that COVID-19 was the underlying cause. Further observe that in the younger age groups, where vaccination started later, excess mortality also starts later.

From the perspective of pharmacovigilance, the simultaneous onset of excess mortality and vaccinations represents a safety signal. Safety signals such as the observation of a temporal relationship between the administration of vaccines and the occurrence of adverse events do not necessarily imply a causal relationship since there may be potential third variables that influence both the course of vaccinations and the course of excess deaths. Thus, a safety signal does not indicate a causal relationship between a side effect and a drug but is only a hypothesis that calls for further assessment.

In fact, in a study by a research team led by Schirmacher [28], out of 35 bodies found unexpectedly dead at home with unclear causes of death within 20 days following COVID vaccination, autopsies revealed causes of death due to pre-existing illnesses in only 10 cases. From the remaining 25 cases, in three cases, it was concluded from the autopsies that vaccination-induced myocarditis was the likely cause of death, and in two cases, it was concluded that vaccination-induced myocarditis was possibly the cause of death. As shown in Supplementary Table 1 published by Schwab et al. [28], vaccination was the cause of death in further cases as well.

Given the temporal relationship between the increase in vaccinations and excess mortality, it seems surprising that a respective safety signal has not been detected in the pharmacovigilance by the Paul-Ehrlich-Institut (PEI), which is responsible for the safety monitoring of drugs in Germany. A closer inspection of the methods used by the PEI to monitor possibly deadly side effects of the COVID-19 vaccinations [29] reveals that a flawed safety analysis is used that will not indicate a safety signal even if a vaccine causes extremely large numbers of unexpected deaths.

The PEI uses a so-called observed-versus-expected analysis where the number of deaths that have been reported to the PEI with a suspected connection to a COVID-19 vaccination is compared to the expected number of all-cause deaths in the vaccinated group. If the number of reported suspected vaccine-related deaths is not significantly higher than the number of expected all-cause deaths (including cancer, heart disease, stroke, etc.), the PEI concludes that there is no safety problem. Such a safety analysis is profoundly flawed since the occurrence of safety signals is essentially impossible. Thus, it is not surprising that a safety signal has not been detected in the pharmacovigilance by the PEI.

As the available mortality data do not allow us to determine the expected and observed numbers of deaths for the vaccinated group only, it is impossible to examine what would have been observed if the PEI had applied a correct safety analysis. To at least demonstrate how a proper observed-versus-expected analysis should be done, two time periods can be compared: the time of April 2020 to March 2021 (the first pandemic year) can be used as a rough estimate of the number of excess deaths without vaccinations. This should be compared to the number of excess deaths in the time of April 2021 to March 2022 (the second pandemic year) where large parts of the population were vaccinated. Table 13 contains the results of such an analysis for six age groups.

For all age groups under 80 years, a significant mortality increase is observed in the second pandemic year when large parts of the population were vaccinated. According to the empirical standard deviations for the different age groups, excess mortalities, which are far beyond $2 \hat \sigma$ did not occur in the first pandemic year without vaccinations but only in the second pandemic year with vaccinations (age groups 30-49 and 60-79 years with $\hat \sigma (d_{30-49}) = 427,\ \hat \sigma (d_{60-79}) = 5,088$). The amount of excess mortality observed in the second pandemic year with vaccinations is much higher than the amount of excess mortality in the first pandemic year without vaccinations. And in the last months April 2022 to December 2022, the situation is even getting worse, excess mortality is still steadily increasing. This, on the one hand, contrasts with the expectation that the vaccination should decrease the number of COVID-19 deaths, and on the other hand, it indicates a safety signal.

The only exception is the last age group 80+, wherein in the first year, a larger number of excess deaths was observed than in the second year. However, it has to be taken into account that in this age range, there was a huge mortality deficit from 2019 until October 2020, which was compensated in November, December 2020, and January 2021. Such an effect could not occur a second time within one year. And at the end of the year 2022, the excess mortality in this age group is again incredibly high.

Taken together, one would expect that vaccinating large parts of the population should have reduced excess mortality. The contrary is observed: both excess mortality and the number of stillbirths increased with increased vaccinations. In all age groups below 80 years, excess mortality was higher in the second year and in particular much higher in the third year of the pandemic, where large parts of the population were vaccinated. These observations are surprising and further more detailed investigations from different scientific fields are strongly recommended to rule out that these safety signals occur due to the existence of unknown side effects of the COVID-19 vaccines.

## Conclusions

This study used the state-of-the-art method of actuarial science to estimate the expected number of all-cause deaths and the increase in all-cause mortality for the pandemic years 2020 to 2022 in Germany. In 2020, the observed number of deaths was extremely close to the expected number, but in 2021, the observed number of deaths was far above the expected number in the order of twice the empirical standard deviation, and in 2022, above the expected number even more than four times the empirical standard deviation. The analysis of the age-dependent monthly excess mortality showed that high excess mortality starting from spring 2021 is responsible for the excess mortality in 2021 and 2022. An analysis of the number of stillbirths revealed a similar mortality pattern than observed for the age group between 15 and 80 years.

As a starting point for further investigations explaining these mortality patterns, we compared the excess mortality to the number of reported COVID-19 deaths and the number of COVID-19 vaccinations. This leads to several open questions, the most important being the covariation between the excess mortality, the number of COVID-19 deaths, and the COVID-19 vaccinations.
